# Supplementary material for: Plural molecular and cellular mechanisms of pore domain KCNQ2 encephalopathy
Source: bioRxiv. 2024 Jun 26:2024.01.04.574177. Preprint. [Version 4] doi: 10.1101/2024.01.04.574177 (PMC10802467; doi:10.1101/2024.01.04.574177)
Supplement: Supplement 9 — Figure 1—figure supplement 1. EEG recording including pre-ictal, post-ictal attenuation and recovery of background of seizure excerpted in Figure 1. As labeled, onset was preceded by eyeblink and muscle artifact. The interval of uninterrupted voltage attenuation between the end of the high voltage fast activity to the first epileptiform burst was 61 sec. Interburst length progressively shortened in length, over about 3 min. Settings as in Figure 1. Link to movie F1-S1. Figure 1—figure supplement 2. Examples of awake and sleep EEG background. There is evidence of state change, with more discontinuity during sleep. The awake excerpt shows variable frequency composition, and excess multifocal sharps. Excess discontinuity and sharps indicate dysmaturity, but burst-suppression is not seen. Settings as in Figure 1, except LFF changed to 1 Hz. Figure 2—figure supplement 1. Movie illustrating position of the G256W substitution within the KCNQ2 channel pore turret and its distance to the selectivity filter. Link to movie F2-S1 Figure 2—figure supplement 2. The G256W variant affects a divergent neuronal KCNQ turret structure enabling forming a bonding network linked to the ion selective pore. A-C. Aligned structural models of the extracellular portions of PGDs of KCNQ1, KCNQ4 and KcsA with that of KCNQ2. Single subunits are shown. D. Cartoon of structural model of turret region of KCNQ4 highlighting the predicted hydrogen bonding network. Several bonds are conserved between KCNQ2 and KCNQ4, but the KCNQ4 network has fewer bonds (compare with Figure 2G). E. Cladogram summarizing evolutionary relationships among several voltage-gated potassium channel genes. Gene duplication(s) are indicated by red circles, and are labeled by a common ancestor (or their extant descendant) possessing both duplicate genes. F. Sequence alignments of KCNQ1-5 P loops and flanking S5 and S6 regions reveal relative conservation of KCNQ5, KCNQ4, and KCNQ2 (as in Figure 2C), and divergence of KCNQ1 and [file NIHPP2024.01.04.574177v4-supplement-9.pdf]

bioRxiv preprint doi: https://doi.org/10.1101/2024.01.04.574177; this version posted June 26, 2024. The copyright holder for this preprint (which was not certified by peer review) is the author/funder, who has granted bioRxiv a license to display the preprint in perpetuity. It is made available under aCC-BY 4.0 International license.

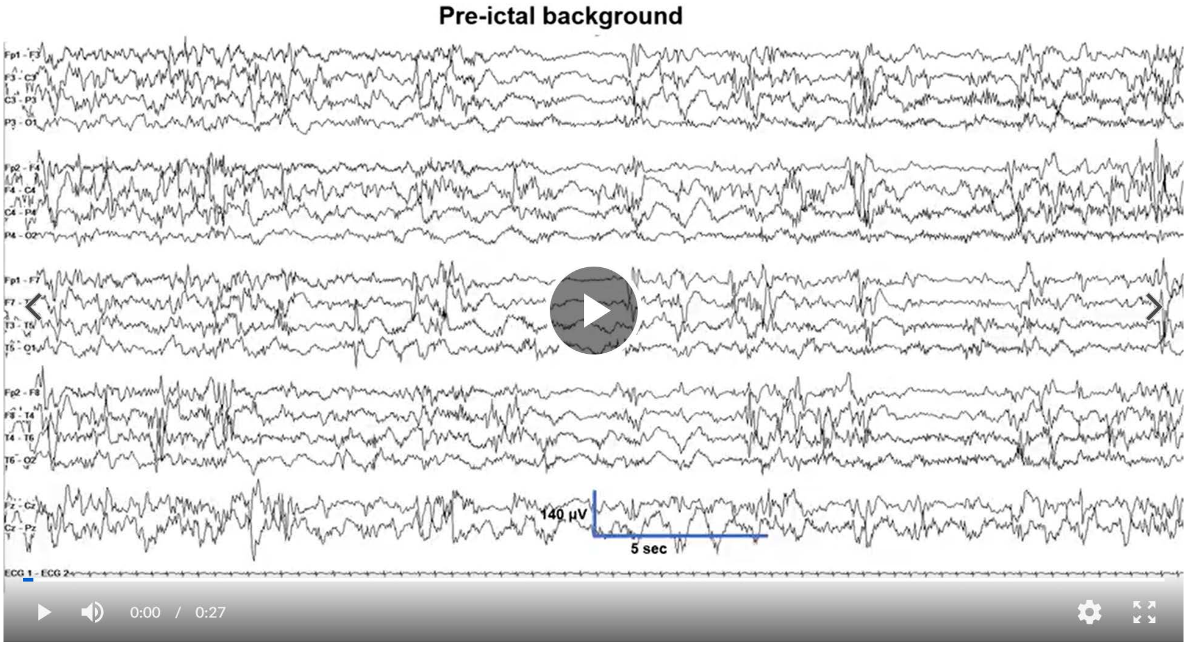

Figure 1—figure supplement 1

bioRxiv preprint doi: https://doi.org/10.1101/2024.01.04.574177; this version posted June 26, 2024. The copyright holder for this preprint (which was not certified by peer review) is the author/funder, who has granted bioRxiv a license to display the preprint in perpetuity. It is made available under aCC-BY 4.0 International license.

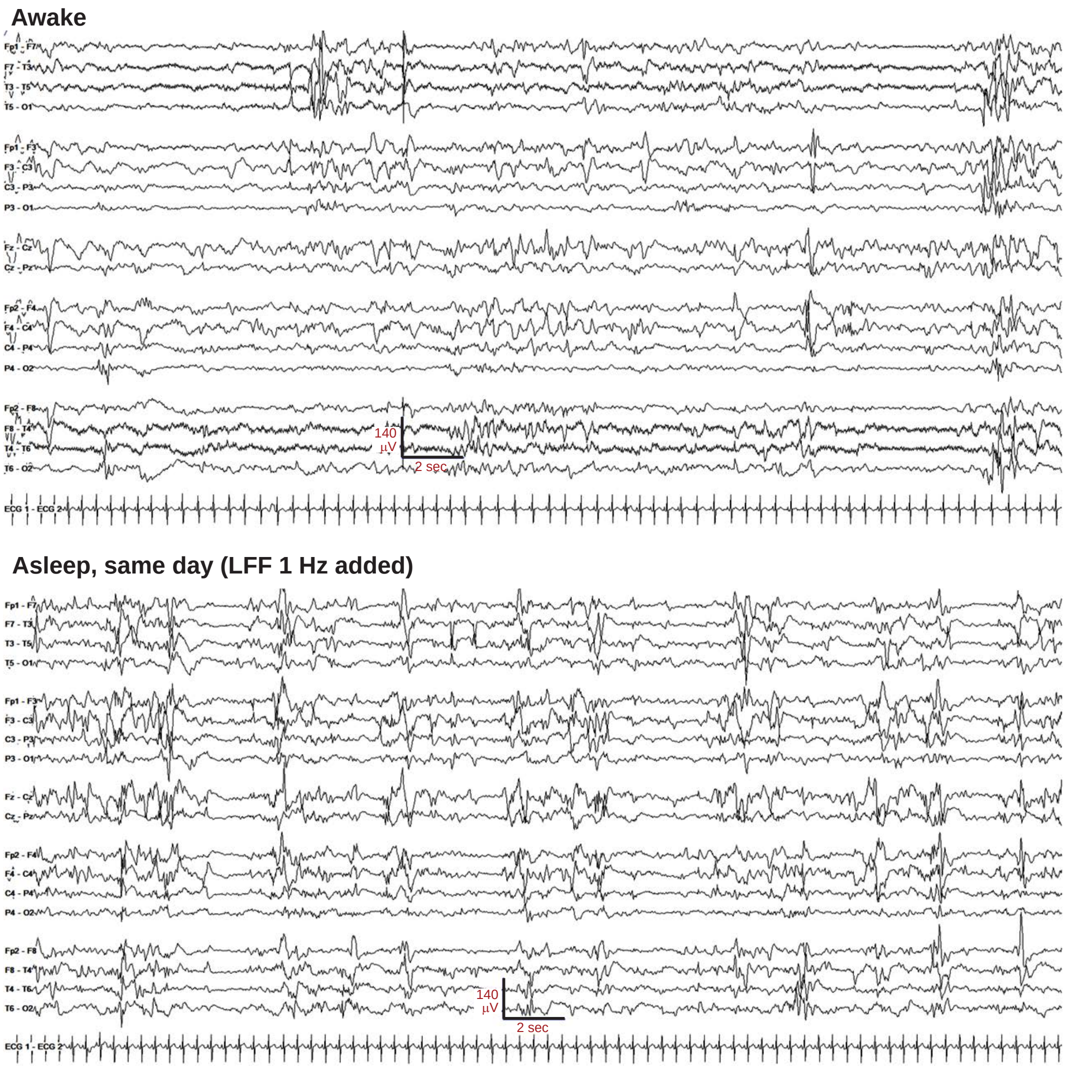

Figure 1—figure supplement 2

bioRxiv preprint doi: https://doi.org/10.1101/2024.01.04.574177; this version posted June 26, 2024. The copyright holder for this preprint (which was not certified by peer review) is the author/funder, who has granted bioRxiv a license to display the preprint in perpetuity. It is made available under aCC-BY 4.0 International license.

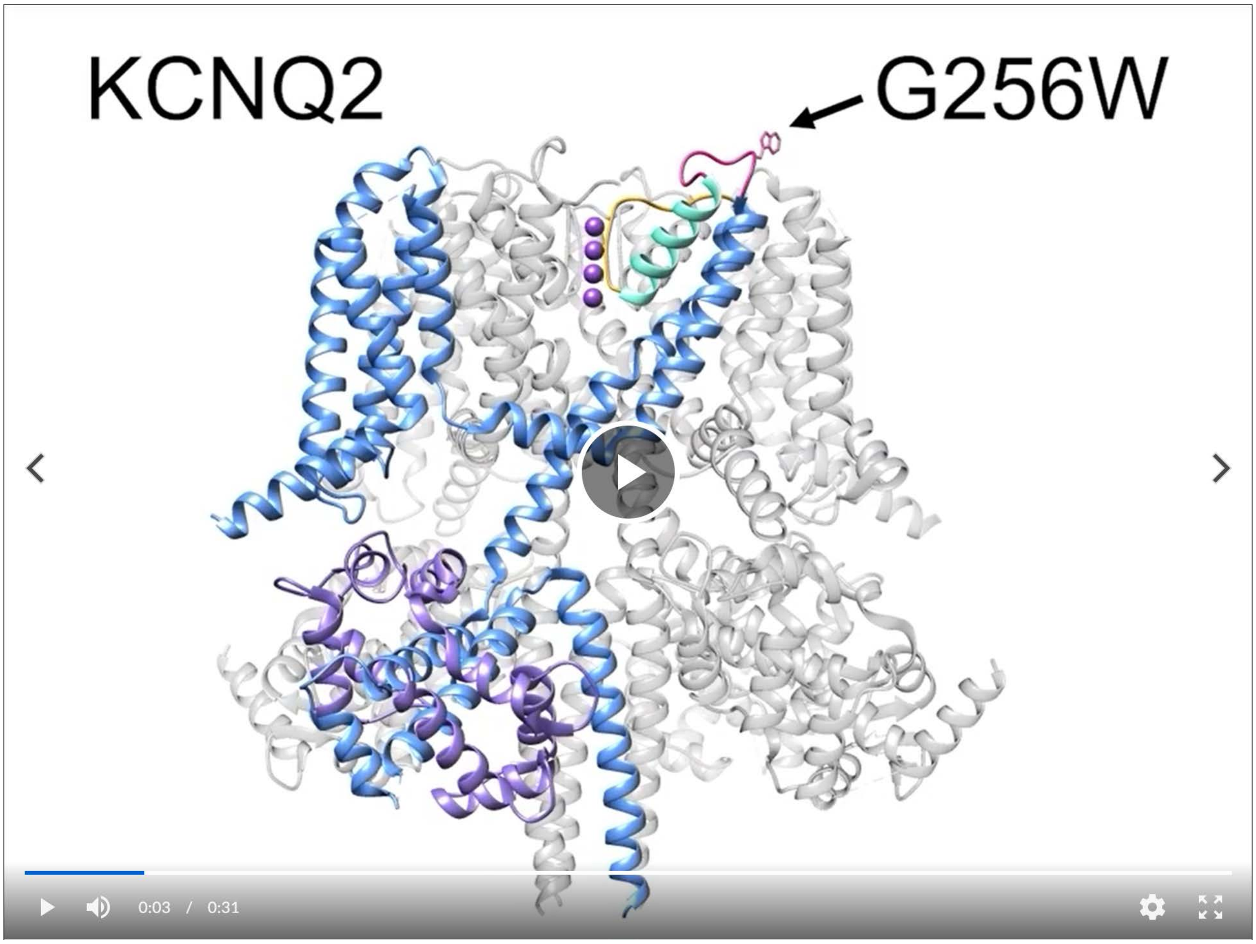

Figure 2—figure supplement 1

bioRxiv preprint doi: https://doi.org/10.1101/2024.01.04.574177; this version posted June 26, 2024. The copyright holder for this preprint (which was not certified by peer review) is the author/funder, who has granted bioRxiv a license to display the preprint in perpetuity. It is made available under aCC-BY 4.0 International license.

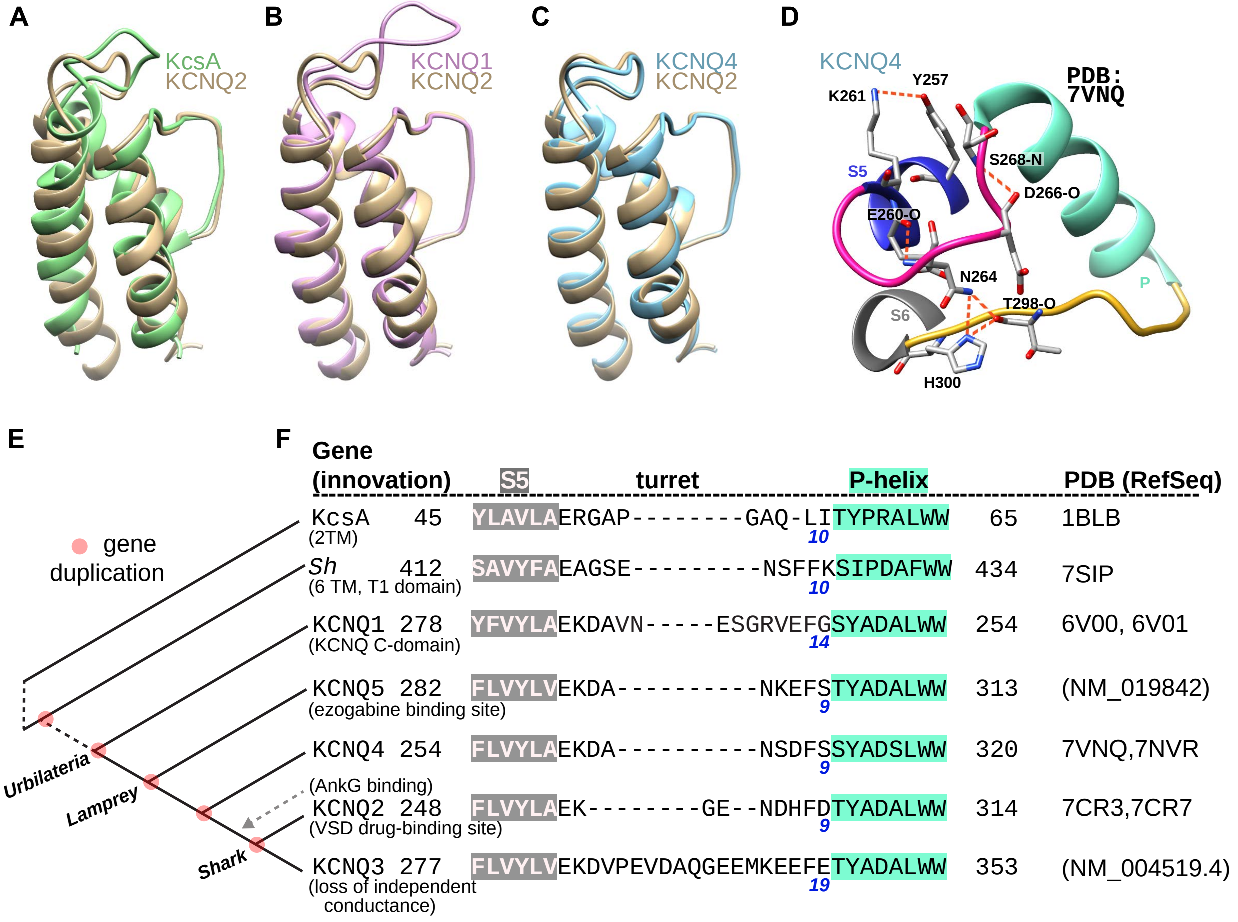

Figure 2—figure supplement 2

bioRxiv preprint doi: https://doi.org/10.1101/2024.01.04.574177; this version posted June 26, 2024. The copyright holder for this preprint (which was not certified by peer review) is the author/funder, who has granted bioRxiv a license to display the preprint in perpetuity. It is made available under aCC-BY 4.0 International license.

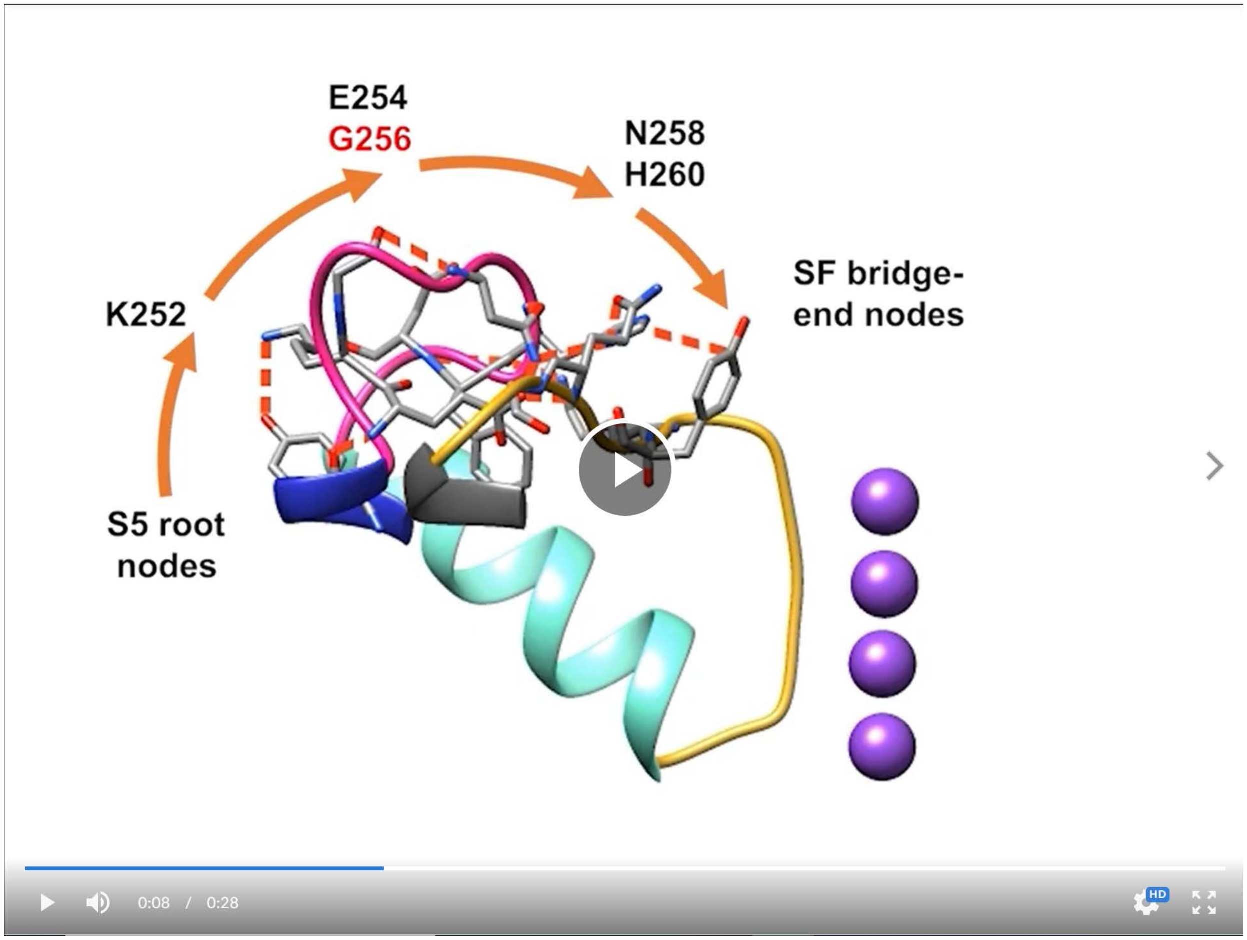

Figure 2—figure supplement 3

bioRxiv preprint doi: https://doi.org/10.1101/2024.01.04.574177; this version posted June 26, 2024. The copyright holder for this preprint (which was not certified by peer review) is the author/funder, who has granted bioRxiv a license to display the preprint in perpetuity. It is made available under aCC-BY 4.0 International license.

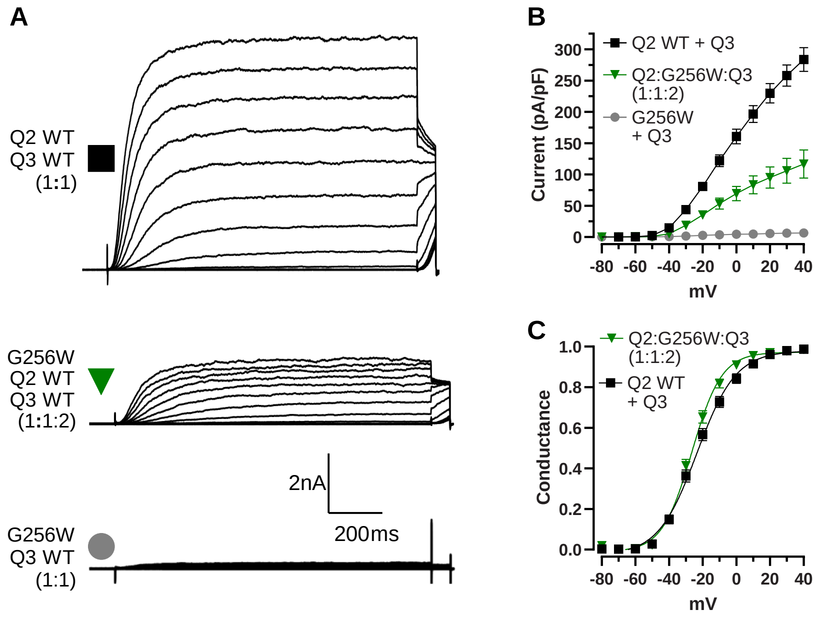

Figure 3—figure supplement 1

bioRxiv preprint doi: https://doi.org/10.1101/2024.01.04.574177; this version posted June 26, 2024. The copyright holder for this preprint (which was not certified by peer review) is the author/funder, who has granted bioRxiv a license to display the preprint in perpetuity. It is made available under aCC-BY 4.0 International license.

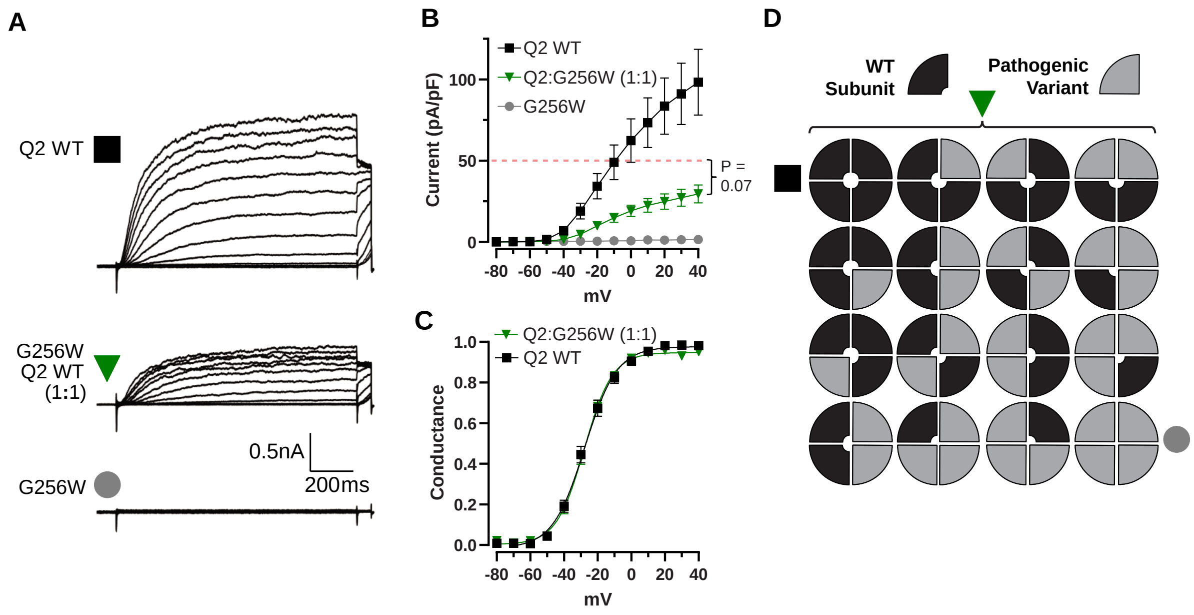

Figure 3—figure supplement 2

bioRxiv preprint doi: https://doi.org/10.1101/2024.01.04.574177; this version posted June 26, 2024. The copyright holder for this preprint (which was not certified by peer review) is the author/funder, who has granted bioRxiv a license to display the preprint in perpetuity. It is made available under aCC-BY 4.0 International license.

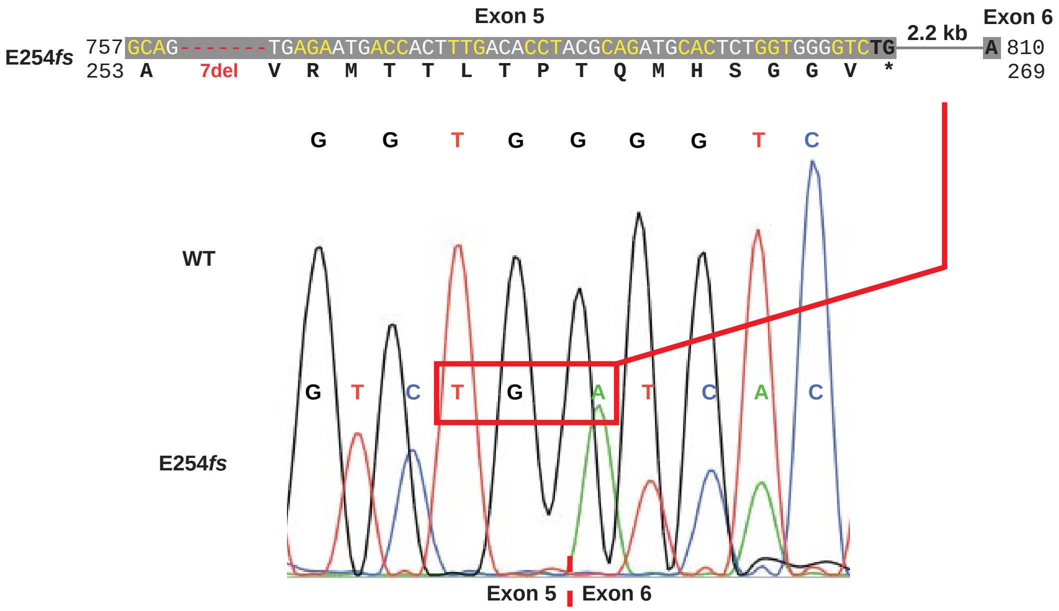

Figure 4—figure supplement 1

bioRxiv preprint doi: https://doi.org/10.1101/2024.01.04.574177; this version posted June 26, 2024. The copyright holder for this preprint (which was not certified by peer review) is the author/funder, who has granted bioRxiv a license to display the preprint in perpetuity. It is made available under aCC-BY 4.0 International license.

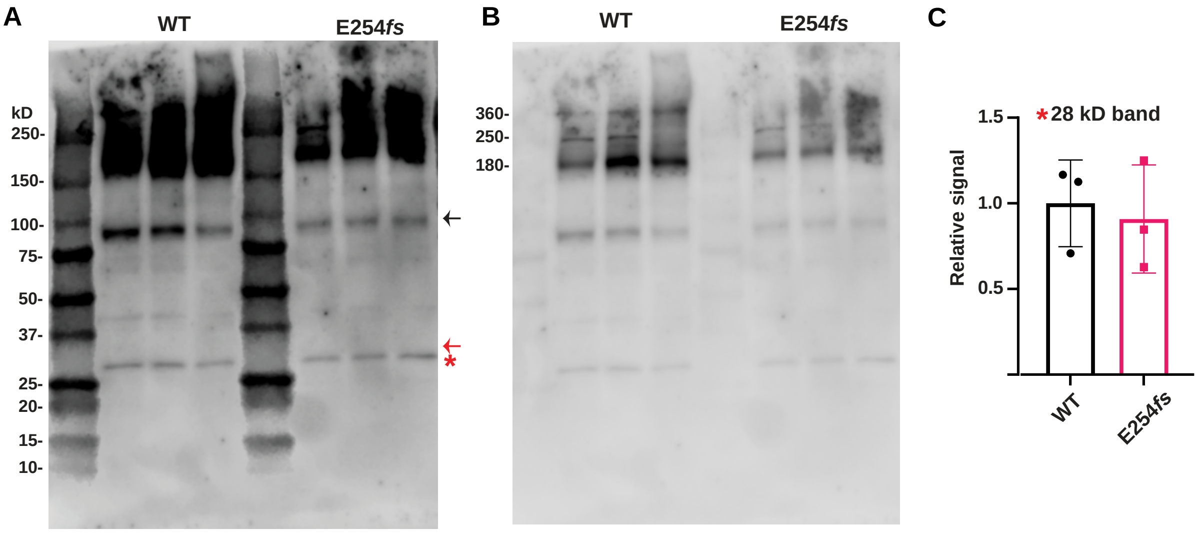

Figure 4—figure supplement 2

bioRxiv preprint doi: https://doi.org/10.1101/2024.01.04.574177; this version posted June 26, 2024. The copyright holder for this preprint (which was not certified by peer review) is the author/funder, who has granted bioRxiv a license to display the preprint in perpetuity. It is made available under aCC-BY 4.0 International license.

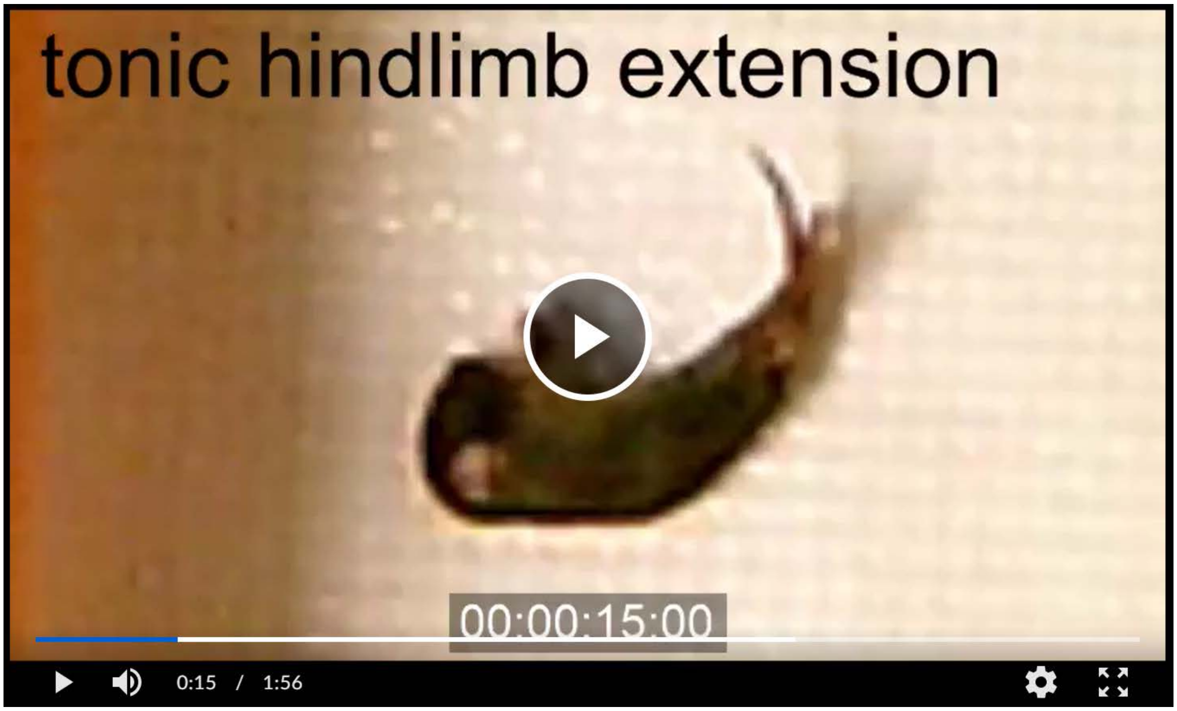

Figure 4—figure supplement 3

bioRxiv preprint doi: https://doi.org/10.1101/2024.01.04.574177; this version posted June 26, 2024. The copyright holder for this preprint (which was not certified by peer review) is the author/funder, who has granted bioRxiv a license to display the preprint in perpetuity. It is made available under aCC-BY 4.0 International license.

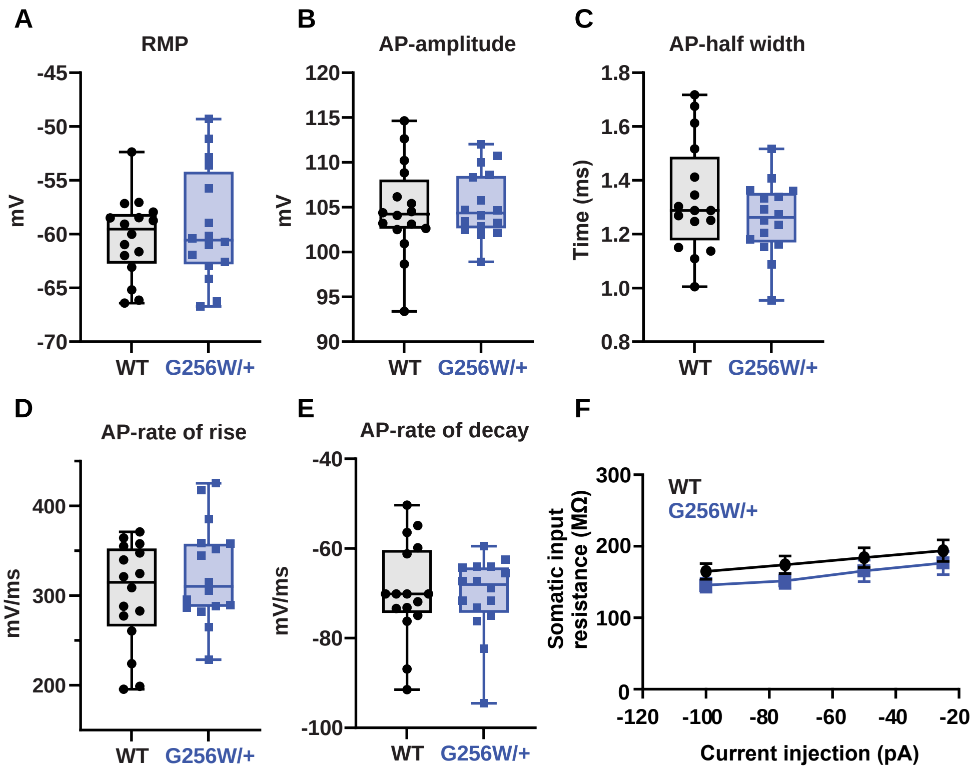

Figure 5—figure supplement 1

bioRxiv preprint doi: https://doi.org/10.1101/2024.01.04.574177; this version posted June 26, 2024. The copyright holder for this preprint (which was not certified by peer review) is the author/funder, who has granted bioRxiv a license to display the preprint in perpetuity. It is made available under aCC-BY 4.0 International license.

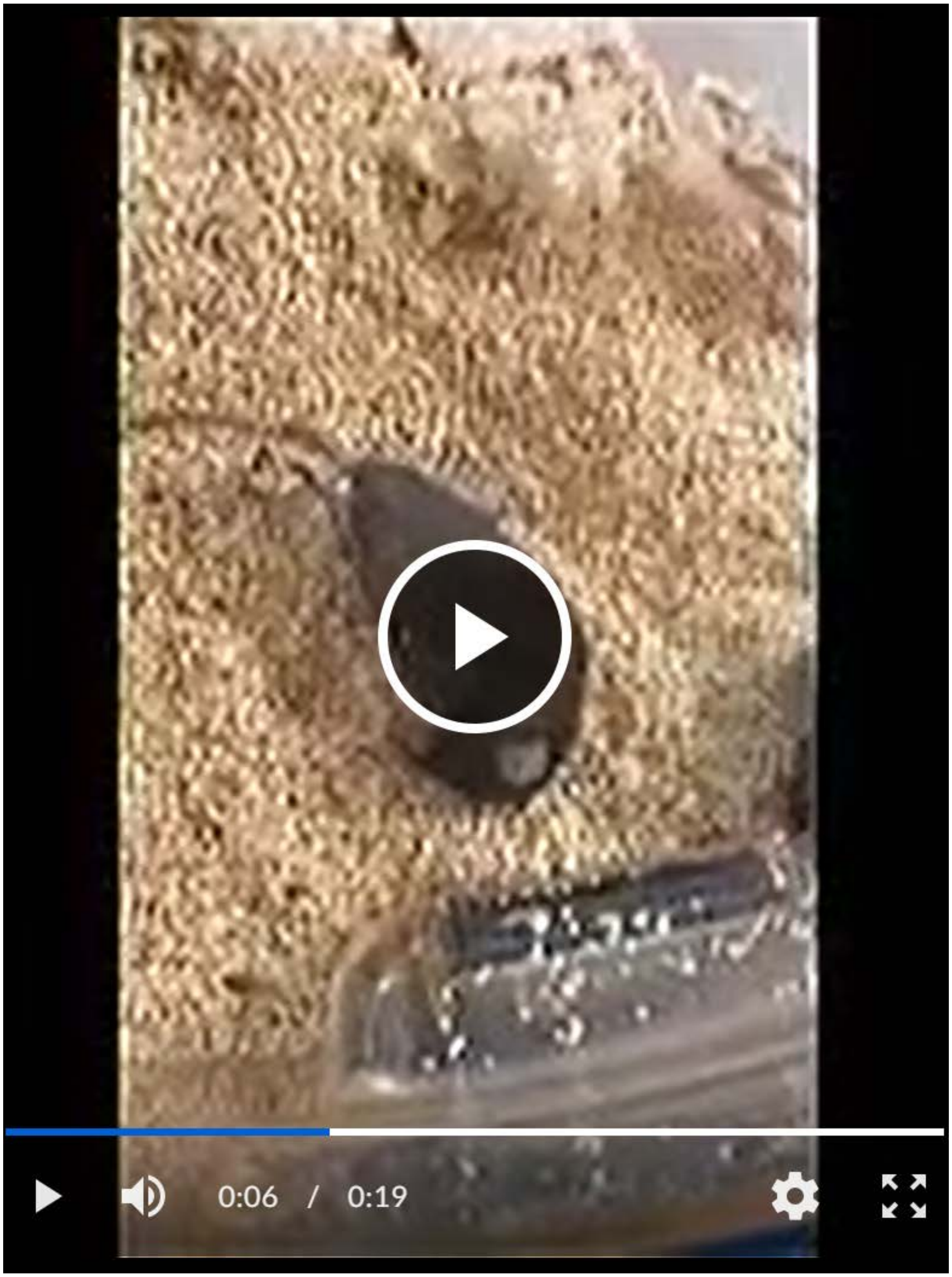

Figure 6—figure supplement 1

bioRxiv preprint doi: https://doi.org/10.1101/2024.01.04.574177; this version posted June 26, 2024. The copyright holder for this preprint (which was not certified by peer review) is the author/funder, who has granted bioRxiv a license to display the preprint in perpetuity. It is made available under aCC-BY 4.0 International license.

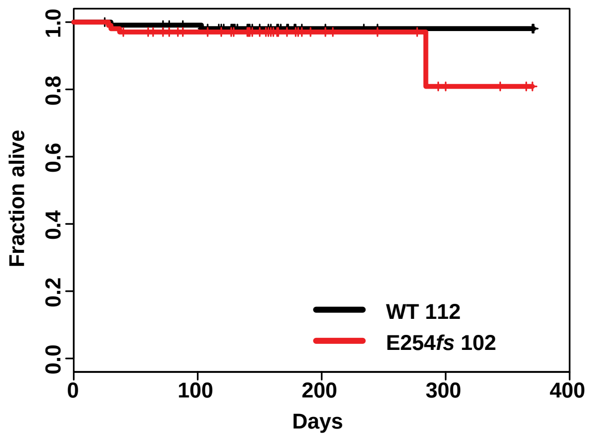

Figure 6—figure supplement 2

bioRxiv preprint doi: https://doi.org/10.1101/2024.01.04.574177; this version posted June 26, 2024. The copyright holder for this preprint (which was not certified by peer review) is the author/funder, who has granted bioRxiv a license to display the preprint in perpetuity. It is made available under aCC-BY 4.0 International license.

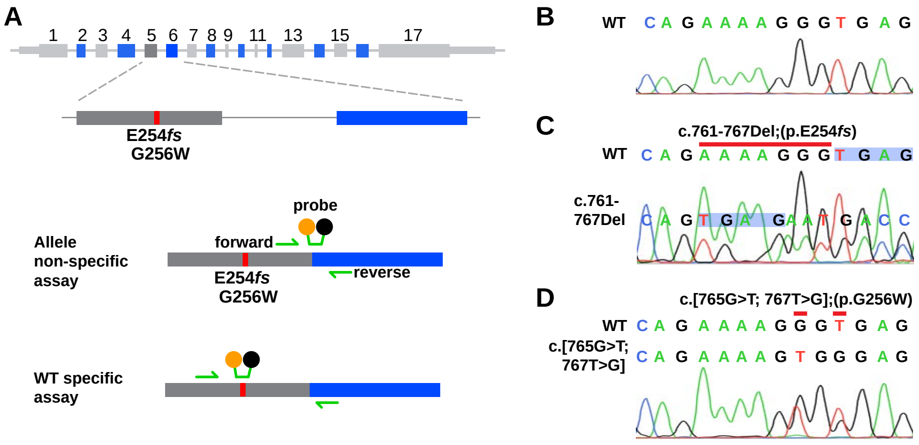

Figure 7—figure supplement 1

bioRxiv preprint doi: https://doi.org/10.1101/2024.01.04.574177; this version posted June 26, 2024. The copyright holder for this preprint (which was not certified by peer review) is the author/funder, who has granted bioRxiv a license to display the preprint in perpetuity. It is made available under aCC-BY 4.0 International license.

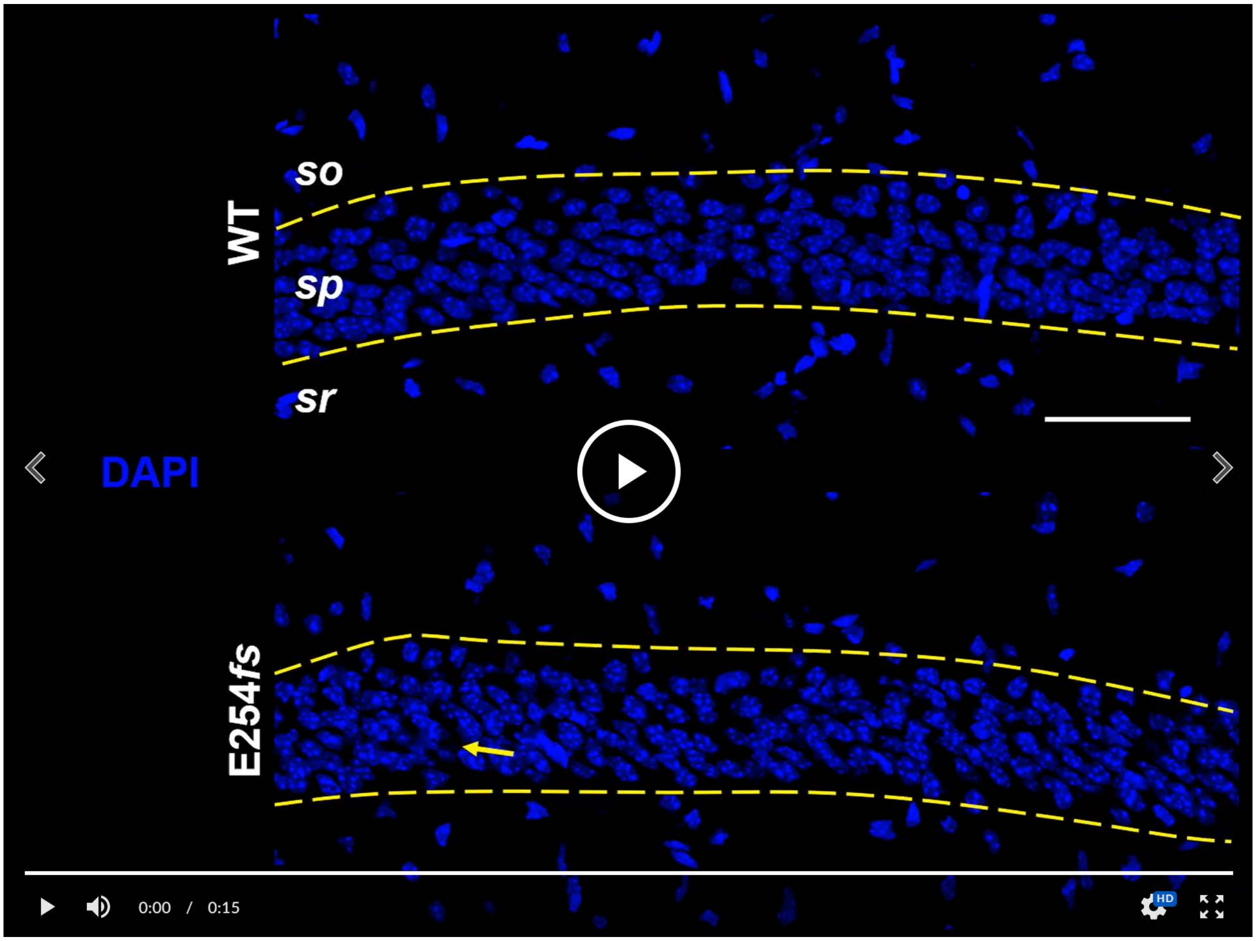

Figure 8—figure supplement 1

bioRxiv preprint doi: https://doi.org/10.1101/2024.01.04.574177; this version posted June 26, 2024. The copyright holder for this preprint (which was not certified by peer review) is the author/funder, who has granted bioRxiv a license to display the preprint in perpetuity. It is made available under aCC-BY 4.0 International license.

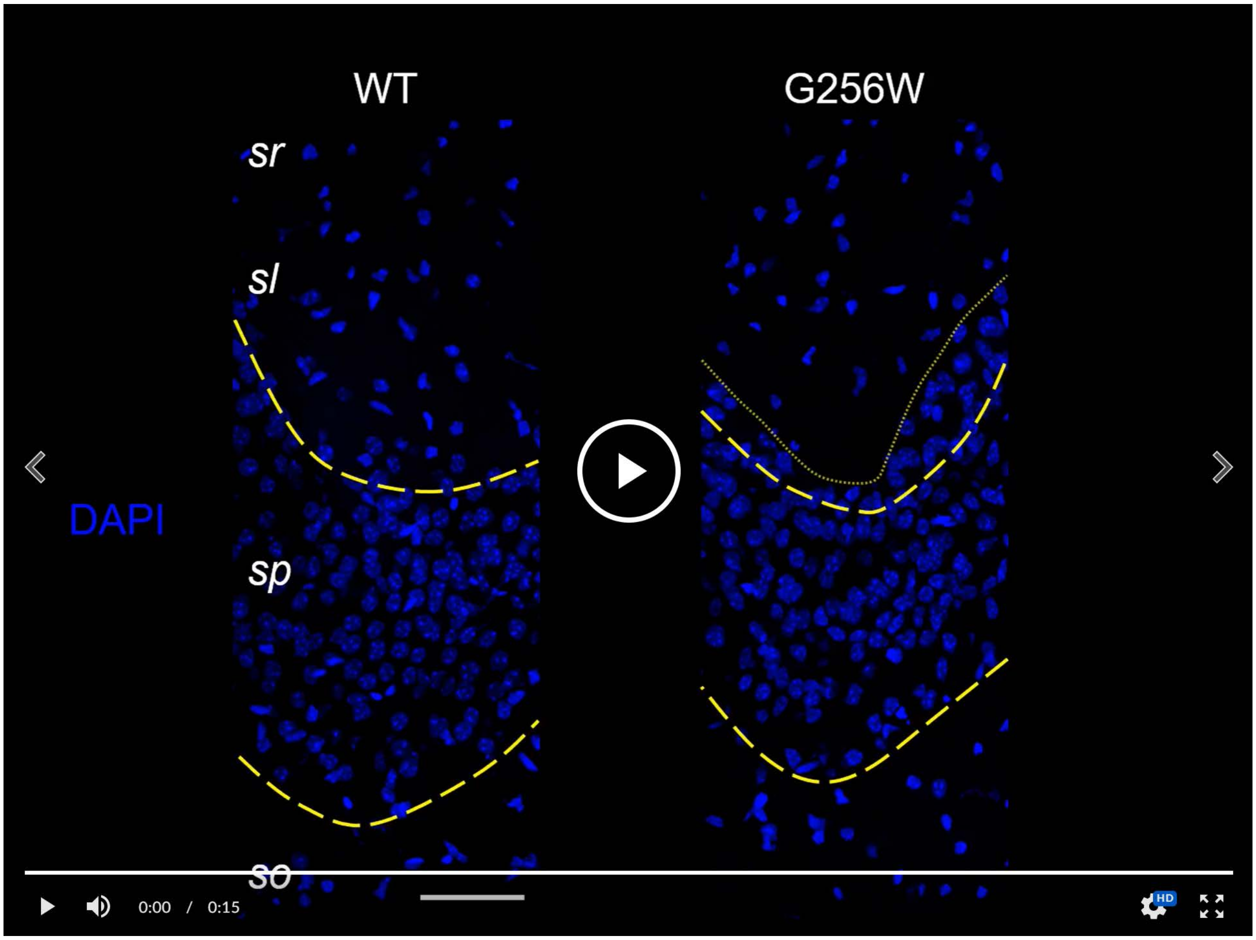

Figure 8—figure supplement 2

bioRxiv preprint doi: https://doi.org/10.1101/2024.01.04.574177; this version posted June 26, 2024. The copyright holder for this preprint (which was not certified by peer review) is the author/funder, who has granted bioRxiv a license to display the preprint in perpetuity. It is made available under aCC-BY 4.0 International license.

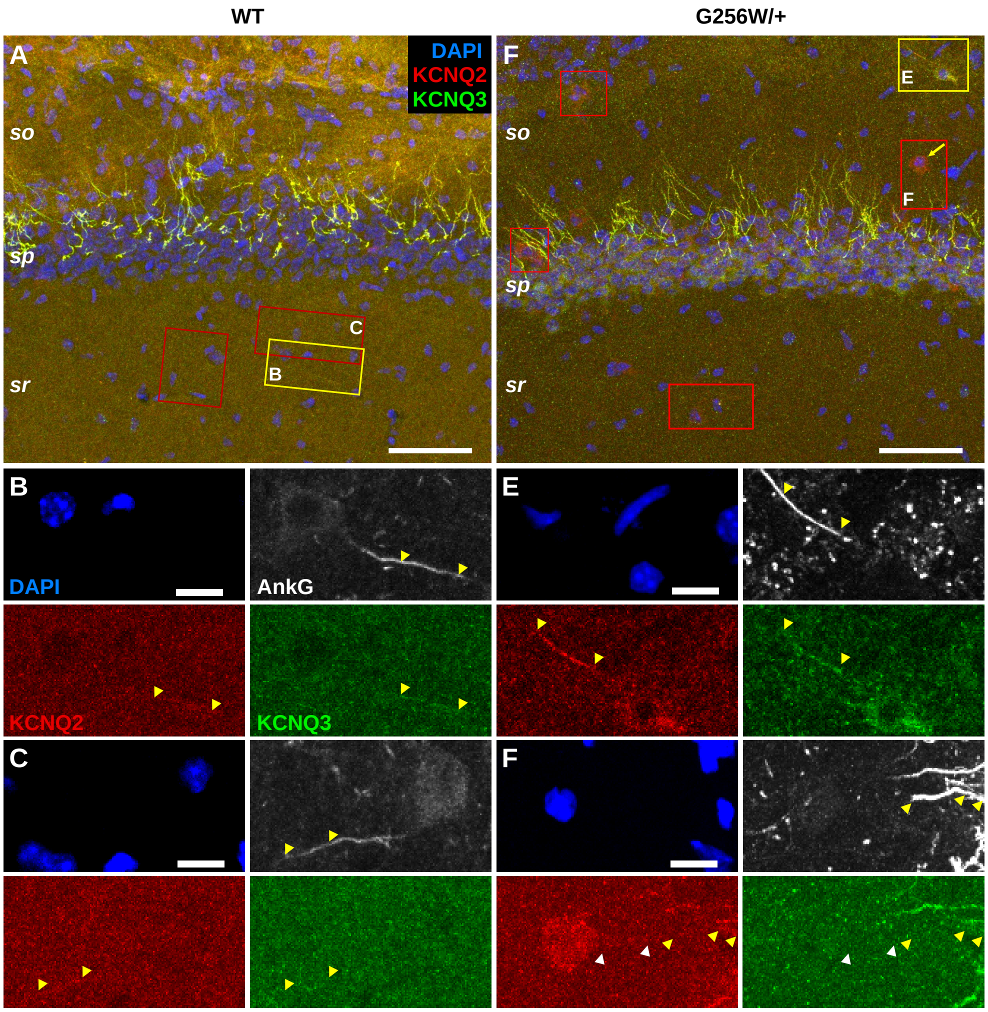

Figure 8—figure supplement 3

bioRxiv preprint doi: https://doi.org/10.1101/2024.01.04.574177; this version posted June 26, 2024. The copyright holder for this preprint (which was not certified by peer review) is the author/funder, who has granted bioRxiv a license to display the preprint in perpetuity. It is made available under aCC-BY 4.0 International license.

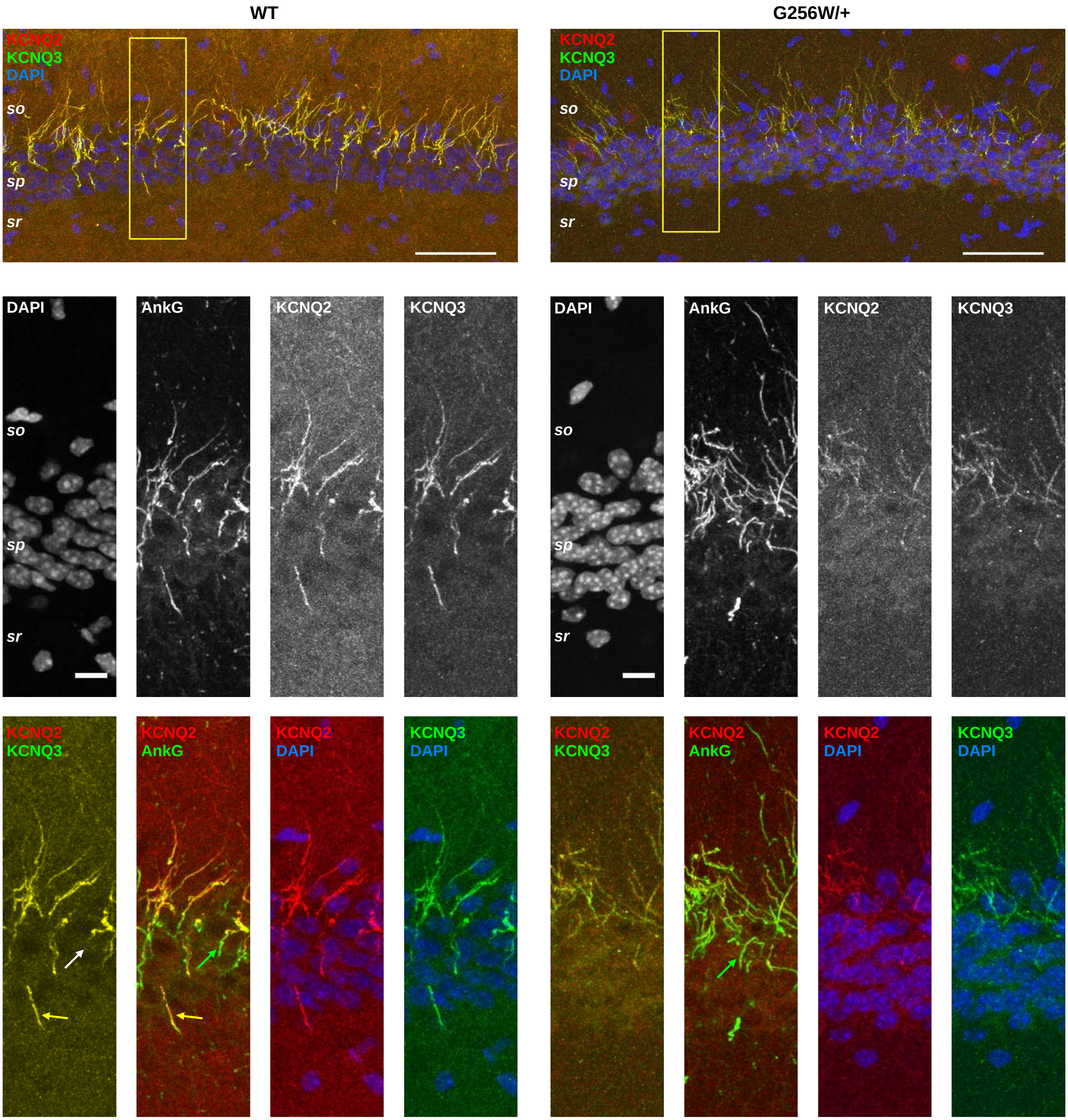

Figure 8—figure supplement 4

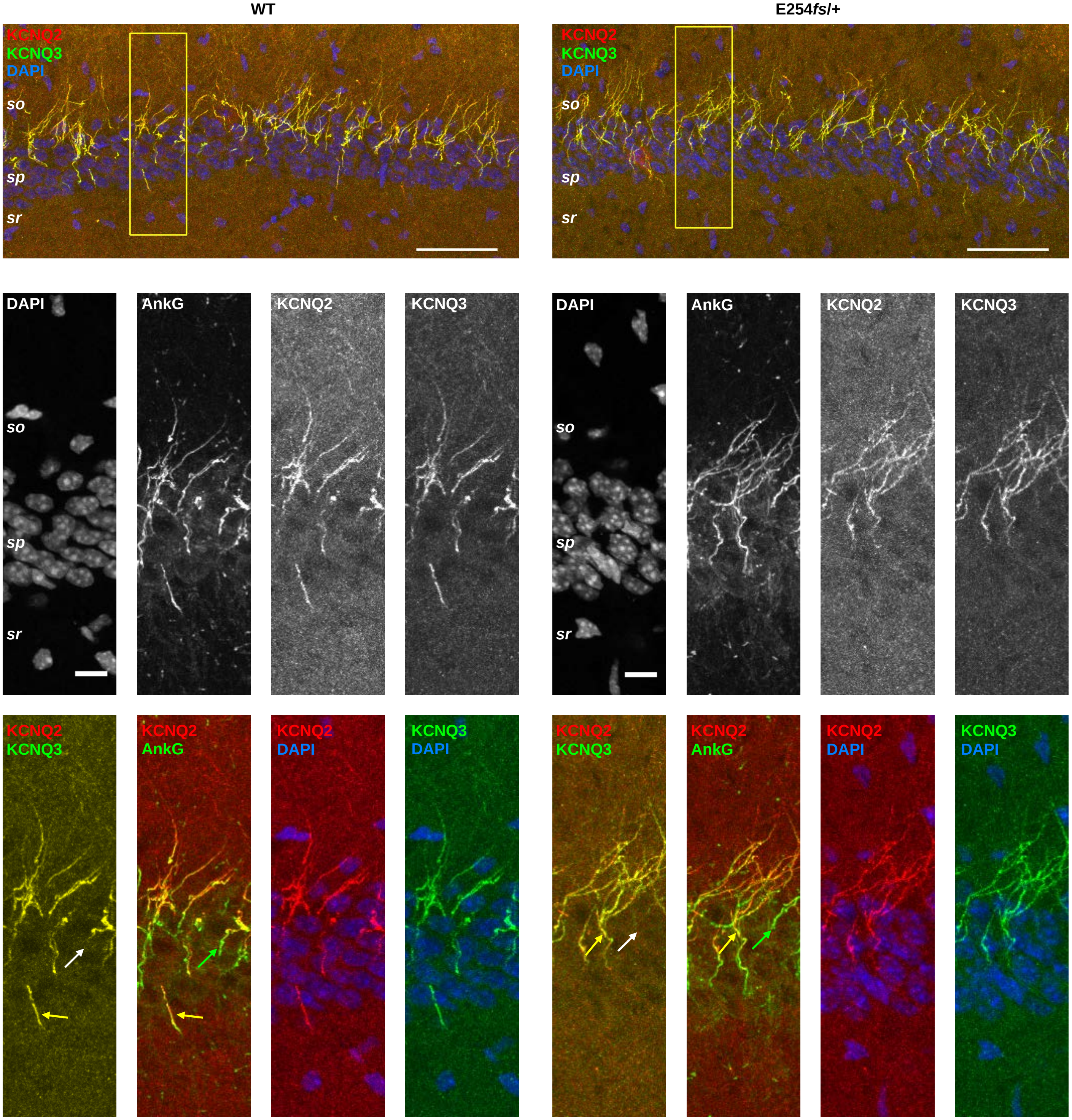

Figure 8—figure supplement 5

bioRxiv preprint doi: https://doi.org/10.1101/2024.01.04.574177; this version posted June 26, 2024. The copyright holder for this preprint (which was not certified by peer review) is the author/funder, who has granted bioRxiv a license to display the preprint in perpetuity. It is made available under aCC-BY 4.0 International license.

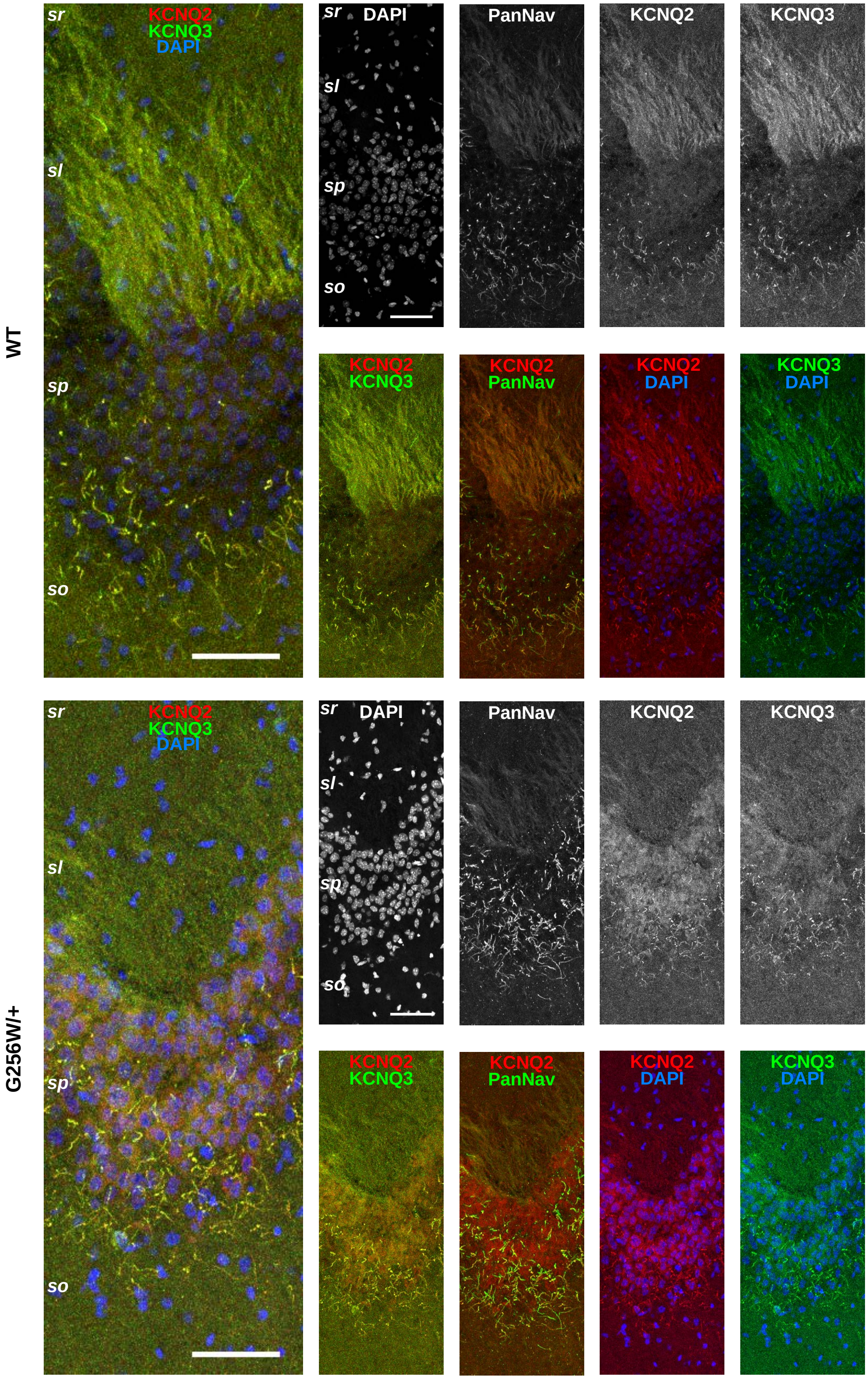

Figure 8—figure supplement 6

bioRxiv preprint doi: https://doi.org/10.1101/2024.01.04.574177; this version posted June 26, 2024. The copyright holder for this preprint (which was not certified by peer review) is the author/funder, who has granted bioRxiv a license to display the preprint in perpetuity. It is made available under aCC-BY 4.0 International license.

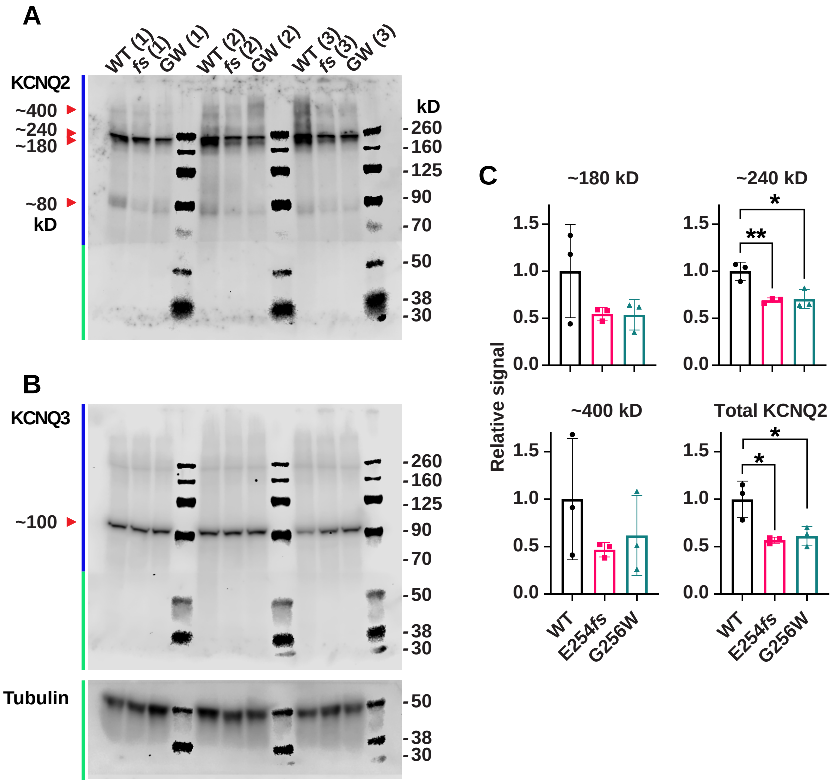

Figure 10—figure supplement 1
