# Supplementary material for: Plural molecular and cellular mechanisms of pore domain KCNQ2 encephalopathy
Source: bioRxiv. 2024 Jan 11:2024.01.04.574177. Preprint. [Version 2] doi: 10.1101/2024.01.04.574177 (PMC10802467; doi:10.1101/2024.01.04.574177)
Supplement: Supplementary file 1 [file NIHPP2024.01.04.574177v2-supplement-1.pdf]

Figure 1—figure supplement 1. EEG recording including pre-ictal, post-ictal attenuation and recovery of background of seizure excerpted in Figure 1. As labeled, onset was preceded by eyeblink and muscle artifact. The interval of uninterrupted voltage attenuation between the end of the high voltage fast activity to the first epileptiform burst was 61 sec. Interburst length progressively shortened in length, over about 3 min. Settings as in Figure 1. Link to movie F1-S1.

Figure 1—figure supplement 2. Awake and sleep EEG background. Settings as in Figure 1, except LFF changed to 1 Hz.

bioRxiv preprint doi: https://doi.org/10.1101/2024.01.04.574177; this version posted January 11, 2024. The copyright holder for this preprint (which was not certified by peer review) is the author/funder, who has granted bioRxiv a license to display the preprint in perpetuity. It is made available under aCC-BY 4.0 International license.

Figure 2—figure supplement 1. Movie illustrating position of the G256W substitution within the KCNQ2 channel pore turret and its distance to the selectivity filter. Link to movie F2-S1

Figure 2—figure supplement 2. Movie illustrating locations of residues contributing to a non-covalent bonding network extending from S5 to the selectivity filter. Link to movie F2-S2

Figure 2—figure supplement 3. The G256W variant affects a divergent neuronal KCNQ turret structure enabling forming a bonding network linked to the ion selective pore. **A-C.** Aligned structural models of the extracellular portions of PGDs of KCNQ1, KCNQ4 and KcsA with that of KCNQ2. Single subunits are shown. **D.** Cartoon of structural model of turret region of KCNQ4 highlighting the predicted hydrogen bonding network. Several bonds are conserved between KCNQ2 and KCNQ4, but the KCNQ4 network has fewer bonds (compare with Figure 2G). **E.** Cladogram summarizing evolutionary relationships among several voltage-gated potassium channel genes. Gene duplication(s) are indicated by red circles, and are labeled by a common ancestor (or their extant descendant) possessing both duplicate genes. **F.** Sequence alignments of KCNQ1-5 P loops and flanking S5 and S6 regions reveal relative conservation of KCNQ5, KCNQ4, and KCNQ2 (as in Figure 2C), and divergence of KCNQ1 and KCNQ3.

bioRxiv preprint doi: https://doi.org/10.1101/2024.01.04.574177; this version posted January 11, 2024. The copyright holder for this preprint (which was not certified by peer review) is the author/funder, who has granted bioRxiv a license to display the preprint in perpetuity. It is made available under aCC-BY 4.0 International license.

Figure 3—figure supplement 1. KCNQ2 G256W co-expression suppresses current in KCNQ2/KCNQ3 heteromeric channels recorded by manual patch-clamp. A. Representative current families for the indicated ratios of subunits. Note currents are larger than in Figure 3. **B-C.** Current/voltage and conductance/voltage relationships for the indicated WT only and G256W/WT cells.

Figure 3—figure supplement 2. KCNQ2 G256W co-expression suppresses current in KCNQ2 homomeric channels recorded by manual patch-clamp. A. Representative current families for the indicated ratios of subunits. Homomeric currents are smaller than in Figure 3—figure supplement 1. **B-C.** Current/voltage and conductance/voltage relationships for the indicated WT only and G256W/WT cells. **D.** Cartoon showing the expected combinations of WT and G256W subunits under heterozygosity based on a simple random association model. Mutant subunits are included in 15/16 of channel tetramers.

bioRxiv preprint doi: https://doi.org/10.1101/2024.01.04.574177; this version posted January 11, 2024. The copyright holder for this preprint (which was not certified by peer review) is the author/funder, who has granted bioRxiv a license to display the preprint in perpetuity. It is made available under aCC-BY 4.0 International license.

Figure 4—figure supplement 1. DNA, RNA, and predicted protein consequences of the G256W and E254*fs16 mutations.** Upper, DNA and predicted protein alignment of the frameshift mutation. Lower, Sanger sequence for cDNA from hippocampal mRNA of an E254*fs*/+ mouse. Splicing occurs at the WT junction, resulting in the predicted in-frame stop codon.

Figure 4—figure supplement 2. Western blotting reveals a lower molecular weight band (Mr ~ 28 kDa) that is equally detected in WT and heterozygous E254*fs*, but no evidence of the E254*fs* truncated protein product. A. Western blot of WT and E254*fs*/+ cortical homogenates (3 biological replicates per genotype, all males), probed with KCNQ2 N-terminal antibody. Black arrow indicates the monomer (Mr ~90kDa), red arrow indicates the estimated relative mobility (~29.73 kDa), of the truncated protein product made from the E254*fs* allele. Red asterisk indicates a ~28 kDa band equally detected in both WT and E254*fs*/+. **B.** Same blot as in **A** but windowed to show higher molecular weight bands. Bands at ~180 kDa and ~360 kDa consistent with predicted mobility of KCNQ2 dimers and tetramers, respectively. A band at ~250 kDa appears in all immunoblots of whole brain homogenates using our KCNQ2 N-terminal antibody. Nano-LC tandem mass spectrometry of peptides from an in-gel digest of this band showed multiple abundant proteins and few KCNQ2 peptides. **C.** Quantification of ~28 kDa band from **A.**

Figure 4—figure supplement 3. Generalized seizure in a P10 heterozygous G256W mouse. This movie includes from 9:01 to 11:05 of a 15:00 min period of open field observation. Animal recovers upright posture at 1:45 in the clip. Link to movie F4-S1

Figure 5—figure supplement 1. AP biophysical properties that are unchanged A. RMP (WT n=16, G256W/+ n=16). **B.** Action potential amplitude (WT n=16, G256W/+ n=16), **C.** Action potential width (WT n=16, G256W/+ n=16), **D.** Action potential rise slope (WT n=16, G256W/+ n=16), **F.** Action potential decay (WT n=16, G256W/+ n=16), and **E.** input resistance (WT n=16, G256W/+ n=16).

bioRxiv preprint doi: https://doi.org/10.1101/2024.01.04.574177; this version posted January 11, 2024. The copyright holder for this preprint (which was not certified by peer review) is the author/funder, who has granted bioRxiv a license to display the preprint in perpetuity. It is made available under aCC-BY 4.0 International license.

Figure 6—figure supplement 1. Video of a fatal convulsive seizure in a 4 month old heterozygous G256W mouse. Seizure onset (not shown) occurred 5-10 sec prior to the start of recording with wild running and jumping, followed by arrest, then resumed (start of video). This was again followed by loss of postural control, followed by sustained forelimb flexor/hindlimb extensor posturing. Attempts to resuscitate the animal were begun immediately and were unsuccessful. Link to movie F6-S1

Figure 6—figure supplement 2. No significant mortality in heterozygous E254*fs* mice. Survival curve of WT vs E254*fs*/+ mice, hashmarks indicate censored mice. E254*fs*/+ mice showed no significant mortality, P = 0.452 Cox propotional hazards model.

bioRxiv preprint doi: https://doi.org/10.1101/2024.01.04.574177; this version posted January 11, 2024. The copyright holder for this preprint (which was not certified by peer review) is the author/funder, who has granted bioRxiv a license to display the preprint in perpetuity. It is made available under aCC-BY 4.0 International license.

Figure 7—figure supplement 1. E254*fs* and G256W variant transcripts are detectable in cDNA from heterozygous animals. A. Upper, WT sequence (bases 758-771). Lower, Sanger trace of amplified *Kcnq2* cDNA from WT hippocampus. **B.** Upper, WT sequence; the red line indicates bases deleted in the E254*fs* allele. Lower, Sanger trace of cDNA from an E254*fs*/+ mouse. Blue shading highlights the shift following the deletion at positions 761-767. Peaks corresponding to E254*fs* transcripts are labeled and are smaller than WT peaks. **C.** Upper, alignment of WT and missense variant DNA sequences, red lines highlight the two base substitutions at codon 256. Below, Sanger trace of amplified cDNA from G256W/+ hippocampus. Double peaks at the mutated bases positions are visible.

Figure 8—figure supplement 1. In CA1, the KCNQ2 and KCNQ3 cellular and subcellular immunolabeling patterns appear similar for WT and heterozygous E254*fs* mice. Ankyrin-G marks position of AISs. KCNQ2 and KCNQ3 strongly label CA1 AISs in E254*fs*/+ mice, and do not show increased somatic labeling compared to WT. Highlighted by an arrow is one interneuron in stratum pyramidale that was somatically labeled for KCNQ2 only. Scale: 50 μm. Link to movie F8-S1

Figure 8—figure supplement 2. Heterozygous G256W mice show increased CA3 pyramidal cell somatic labeling and reduced mossy fiber labeling for KCNQ2 and KCNQ3. Yellow lines demarcate the borders of sp; the sp-sl border is cut obliquely through the tissue section in the G256W/+ sample. PanNav strongly labels the unmylenated axons of the mossy fibers in stratum lucidum of both samples. PanNav also labels the obliquely cut AISs of pyramidal cell neurons, which are mostly located within sp. Scale: 50 μm. Link to movie F8-S2

bioRxiv preprint doi: https://doi.org/10.1101/2024.01.04.574177; this version posted January 11, 2024. The copyright holder for this preprint (which was not certified by peer review) is the author/funder, who has granted bioRxiv a license to display the preprint in perpetuity. It is made available under aCC-BY 4.0 International license.

Figure 8—figure supplement 3. A heterozygous G256W mouse CA1 Stratum oriens interneuron is somatically labeled for both KCNQ2 and KCNQ3. A. Wider view of the same G256W/+ CA1 image stack shown in Figure 8 movie, showing corpus callosum (cc, partial), alveus (alv), so, sp, and a larger portion of sr. Four interneurons somatically labelled for KCNQ2 but not KCNQ3 are enclosed by red boxes. The interneuron indicated with an arrow in Figure 8 movie (KCNQ2 labeled, KCNQ3 unlabeled), is again highlighted with an arrow. The yellow box encloses an interneuron somatically co-labeled for KCNQ2 and KCNQ3. **B.** Individual laser channels for the interneuron enclosed by yellow box in A. The soma is labeled for both KCNQ2 and KCNQ3. The nearby AIS showing AnkG, KCNQ2, and KCNQ3 labeling likely arises from this cell, but its origin was not verified by higher resolution re-imaging. Scales: A: 50 μm, B: 10 μm.

Figure 9—figure supplement 1. KCNQ2 protein is reduced in neocortex of P21 heterozygous E254*fs* and G256W mice. A-B. Representative western blots for all three genotypes probed for KCNQ2 and KCNQ3. **C.** Quantified KCNQ2 signal relative to wildtype. **D.** Quantified KCNQ3 signal relative to wildtype, normalized to protein loaded as assayed by BCA. n=3 per genotype, all males. One way ANOVA, * = $P \leq 0.05$, ** = $P \leq 0.005$, *** = $P \leq 0.0005$.

Figure 9—figure supplement 2. Images of entire filter used for western blot of lysates, probed for KCNQ2 and KCNQ3. A. PVDF filter with electrotransferred brain proteins was cut below the 75 kDa marker. Upper portion was probed for KCNQ2; lower portion was probed for tubulin. Black arrow points to KCNQ2 monomer band (Mr ~90 kDa). Red arrow points to a band at ~250 kDa. Mass spectrometry of in gel digest of this band showed multiple abundant proteins and few KCNQ2 peptides. Also visible are bands likely representing KCNQ2 dimer formation (Mr ~180 kDa and higher molecular weight). **B.** KCNQ3 filter was cut below 75 kDa in order to probe for Tubulin. Black arrow points to predicted KCNQ3 monomer with a Mr ~100 kDa. The red asterisk indicates a bad sample that was not included in the analysis seen in Figure 9—figure supplement

1. **C.** KCNQ2 band intensity normalized to tubulin. **D.** KCNQ3 band intensity signal normalized to Tubulin.

bioRxiv preprint doi: https://doi.org/10.1101/2024.01.04.574177; this version posted January 11, 2024. The copyright holder for this preprint (which was not certified by peer review) is the author/funder, who has granted bioRxiv a license to display the preprint in perpetuity. It is made available under aCC-BY 4.0 International license.

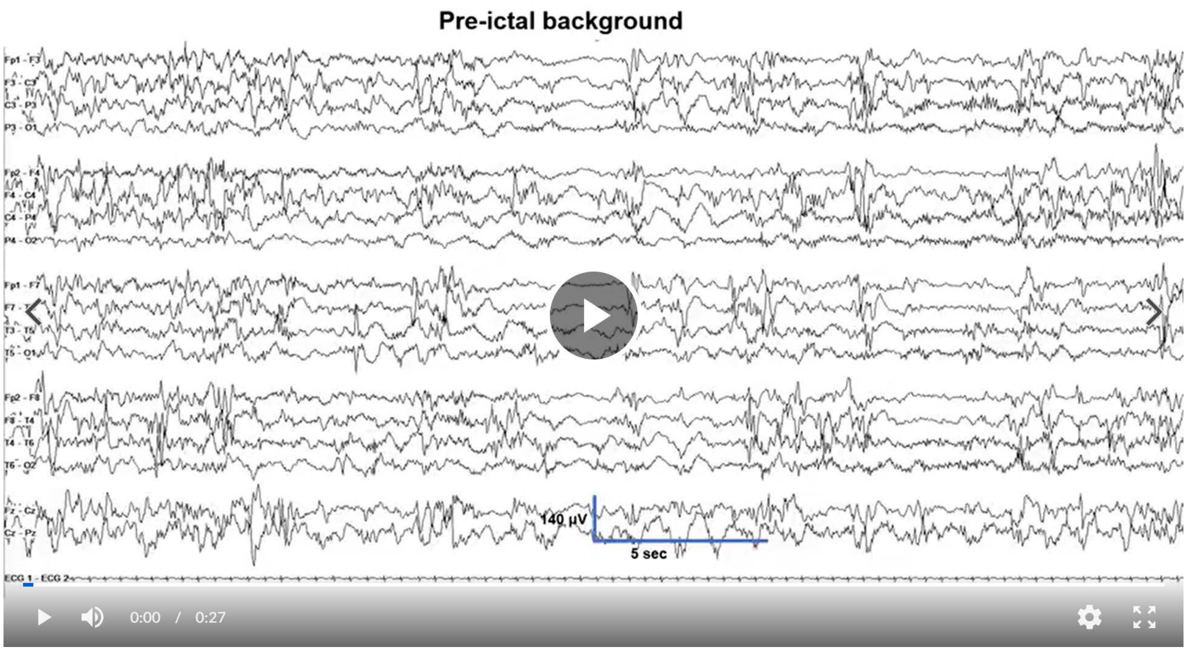

https://bcm.box.com/s/2qj0ctp6mypbx22xbmc4hxfmsxsk78zc

Figure 1—figure supplement 1

bioRxiv preprint doi: https://doi.org/10.1101/2024.01.04.574177; this version posted January 11, 2024. The copyright holder for this preprint (which was not certified by peer review) is the author/funder, who has granted bioRxiv a license to display the preprint in perpetuity. It is made available under aCC-BY 4.0 International license.

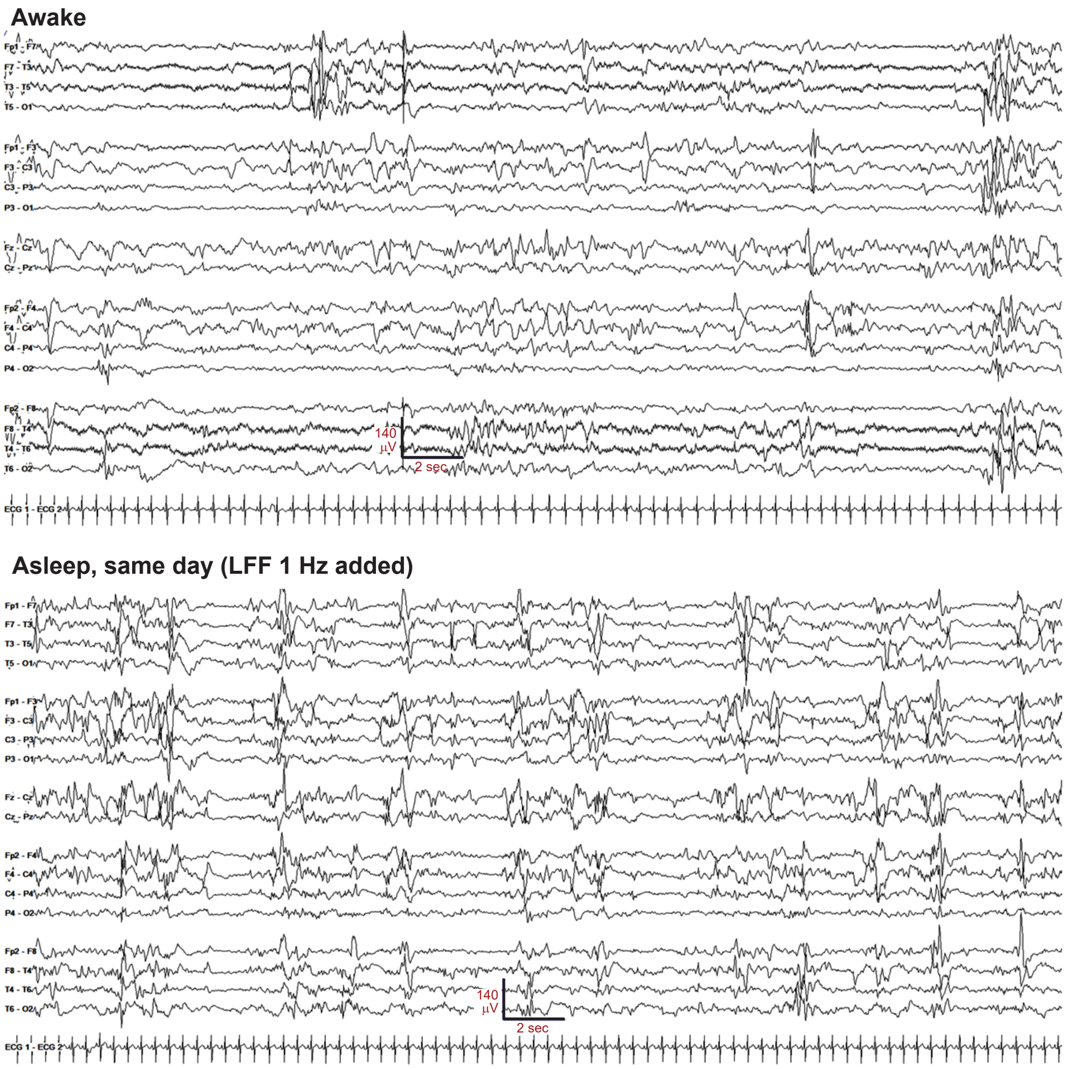

Figure 1—figure supplement 2

bioRxiv preprint doi: https://doi.org/10.1101/2024.01.04.574177; this version posted January 11, 2024. The copyright holder for this preprint (which was not certified by peer review) is the author/funder, who has granted bioRxiv a license to display the preprint in perpetuity. It is made available under aCC-BY 4.0 International license.

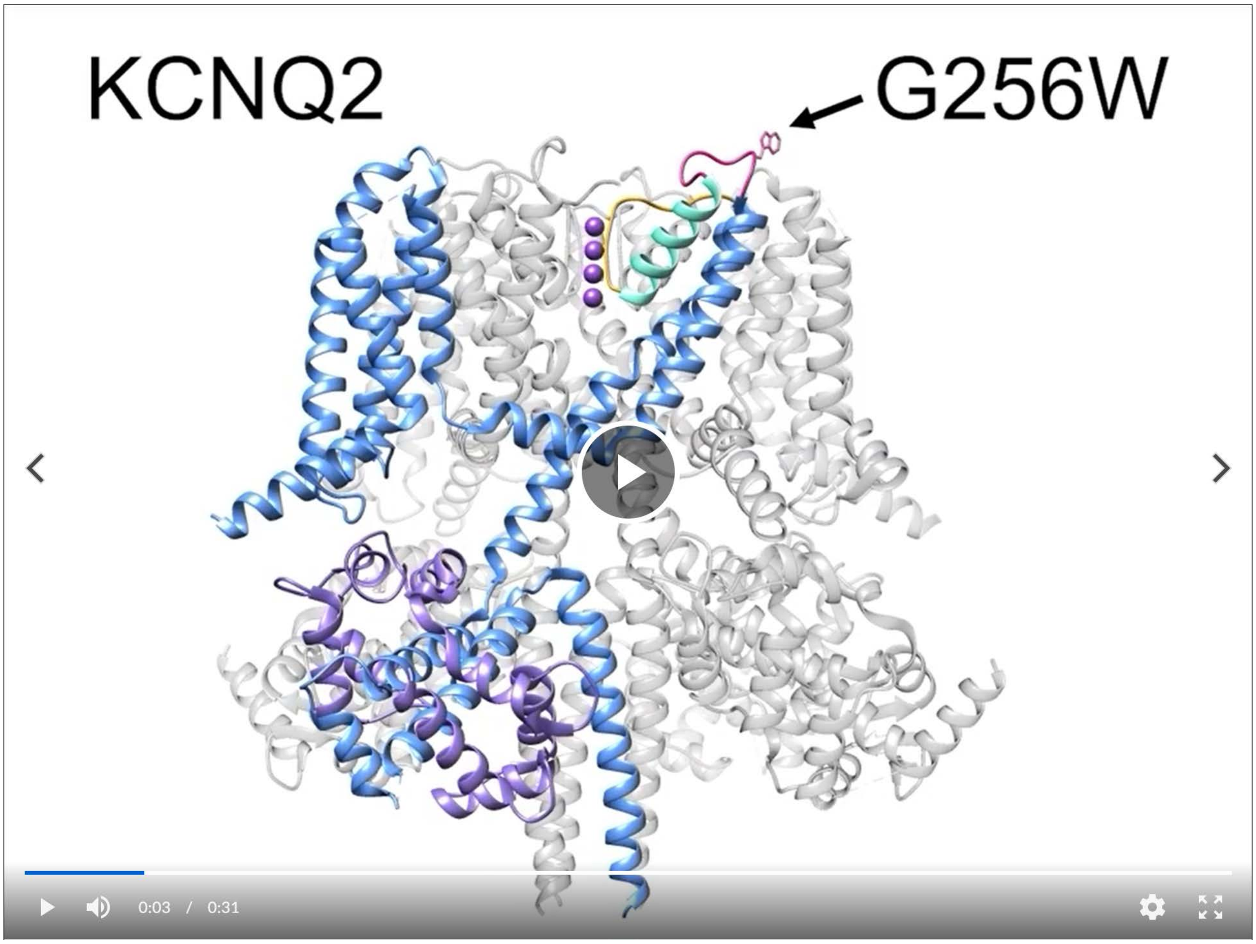

https://bcm.box.com/s/sty5x4nuxgrhe3ql4gpyn9n28uc3qznh

Figure 2—figure supplement 1

bioRxiv preprint doi: https://doi.org/10.1101/2024.01.04.574177; this version posted January 11, 2024. The copyright holder for this preprint (which was not certified by peer review) is the author/funder, who has granted bioRxiv a license to display the preprint in perpetuity. It is made available under aCC-BY 4.0 International license.

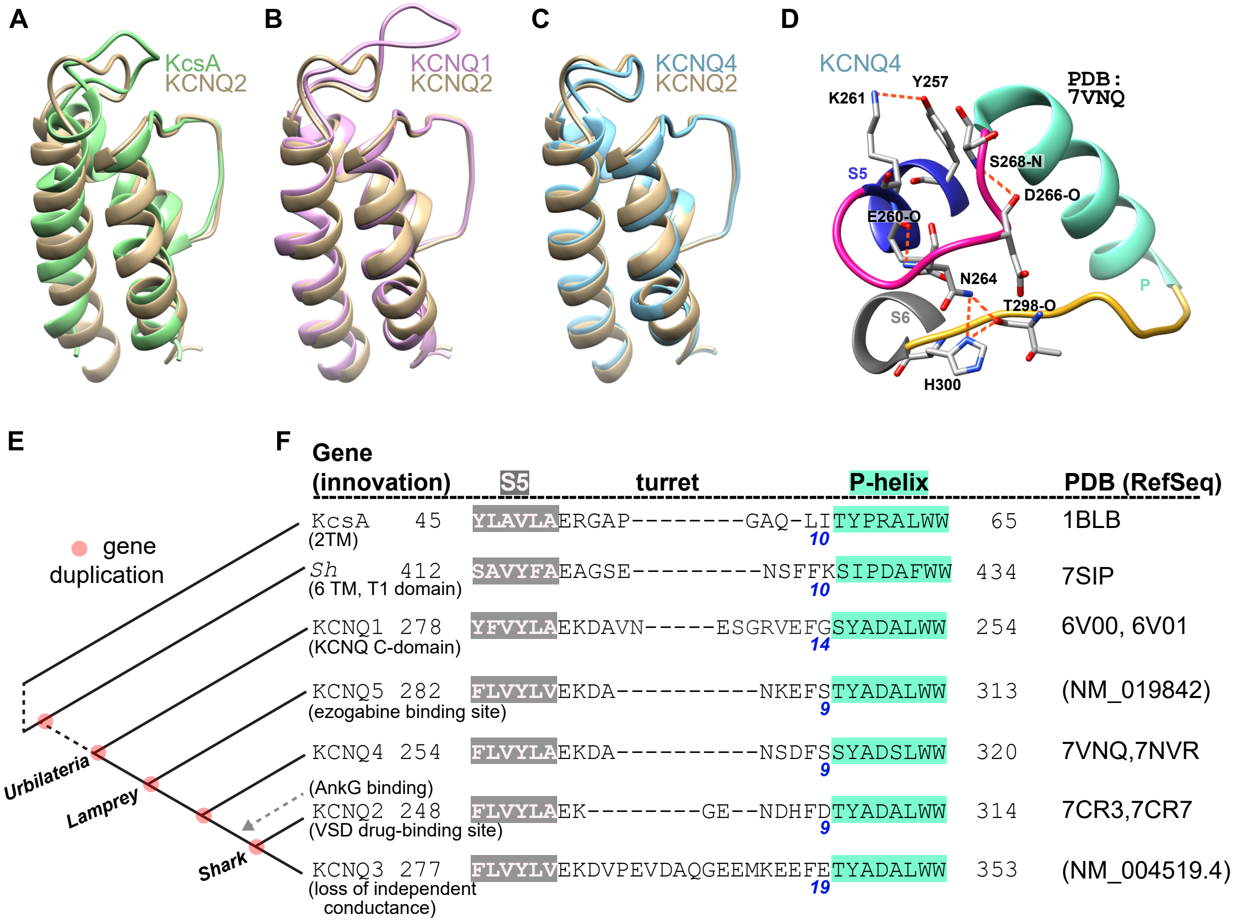

Figure 2—figure supplement 2

bioRxiv preprint doi: https://doi.org/10.1101/2024.01.04.574177; this version posted January 11, 2024. The copyright holder for this preprint (which was not certified by peer review) is the author/funder, who has granted bioRxiv a license to display the preprint in perpetuity. It is made available under aCC-BY 4.0 International license.

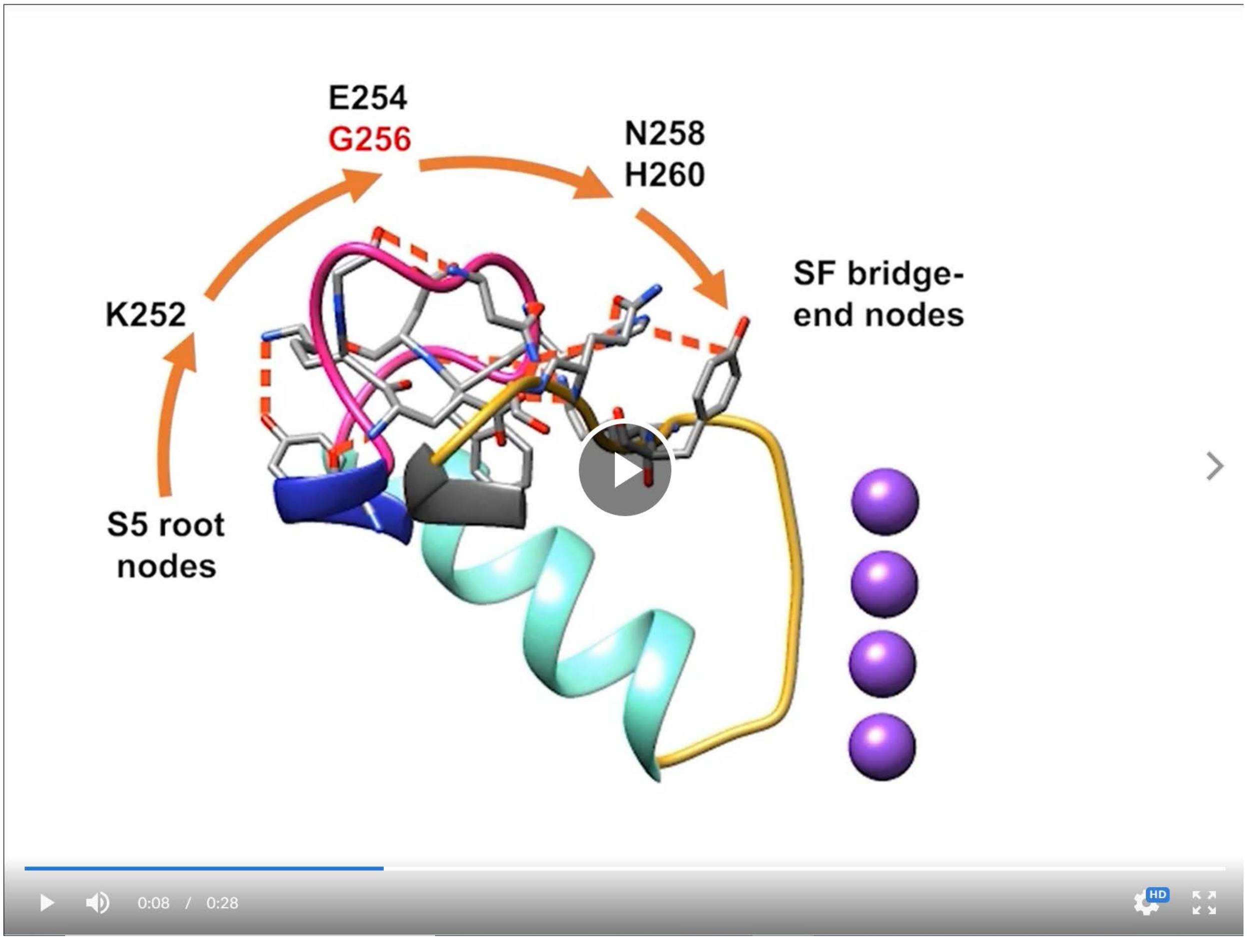

https://bcm.box.com/s/6ej1d4tum74l9mta0yw3rdjubnxoz89n

Figure 2—figure supplement 3

bioRxiv preprint doi: https://doi.org/10.1101/2024.01.04.574177; this version posted January 11, 2024. The copyright holder for this preprint (which was not certified by peer review) is the author/funder, who has granted bioRxiv a license to display the preprint in perpetuity. It is made available under aCC-BY 4.0 International license.

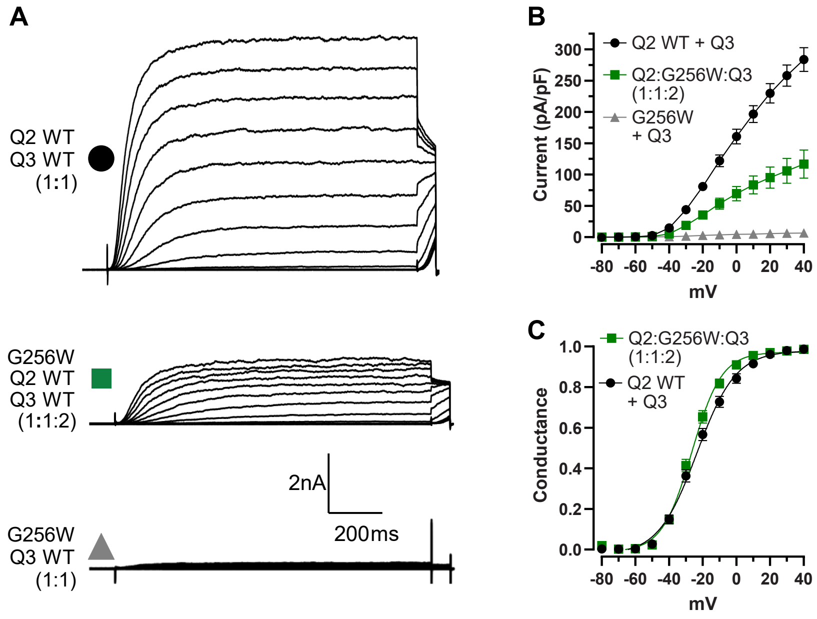

Figure 3—figure supplement 1

bioRxiv preprint doi: https://doi.org/10.1101/2024.01.04.574177; this version posted January 11, 2024. The copyright holder for this preprint (which was not certified by peer review) is the author/funder, who has granted bioRxiv a license to display the preprint in perpetuity. It is made available under aCC-BY 4.0 International license.

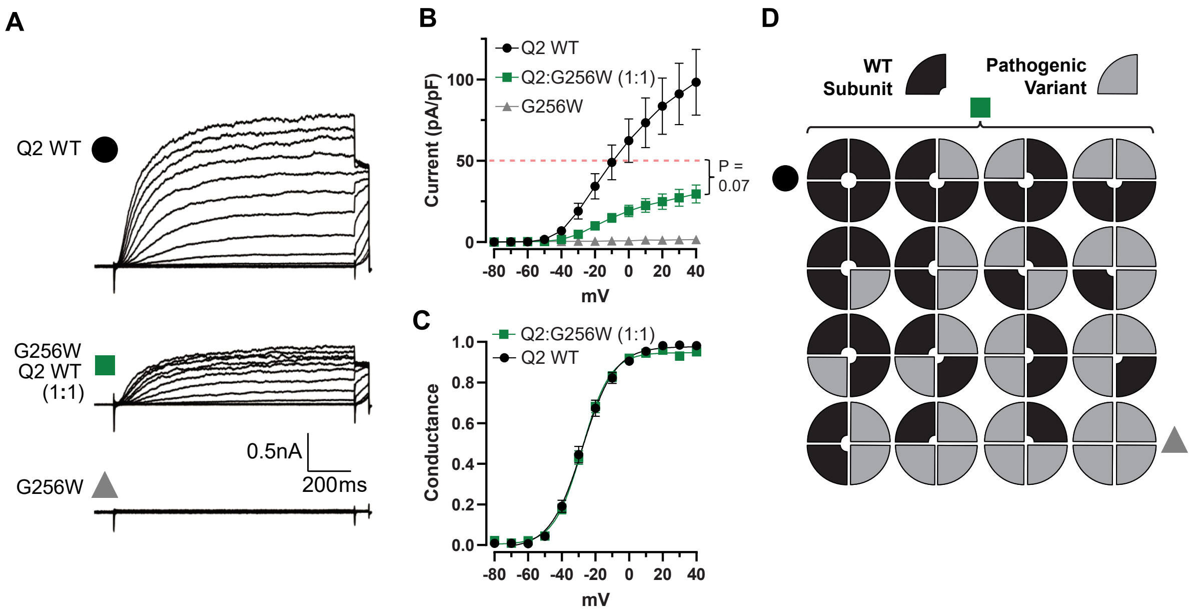

Figure 3—figure supplement 2

bioRxiv preprint doi: https://doi.org/10.1101/2024.01.04.574177; this version posted January 11, 2024. The copyright holder for this preprint (which was not certified by peer review) is the author/funder, who has granted bioRxiv a license to display the preprint in perpetuity. It is made available under aCC-BY 4.0 International license.

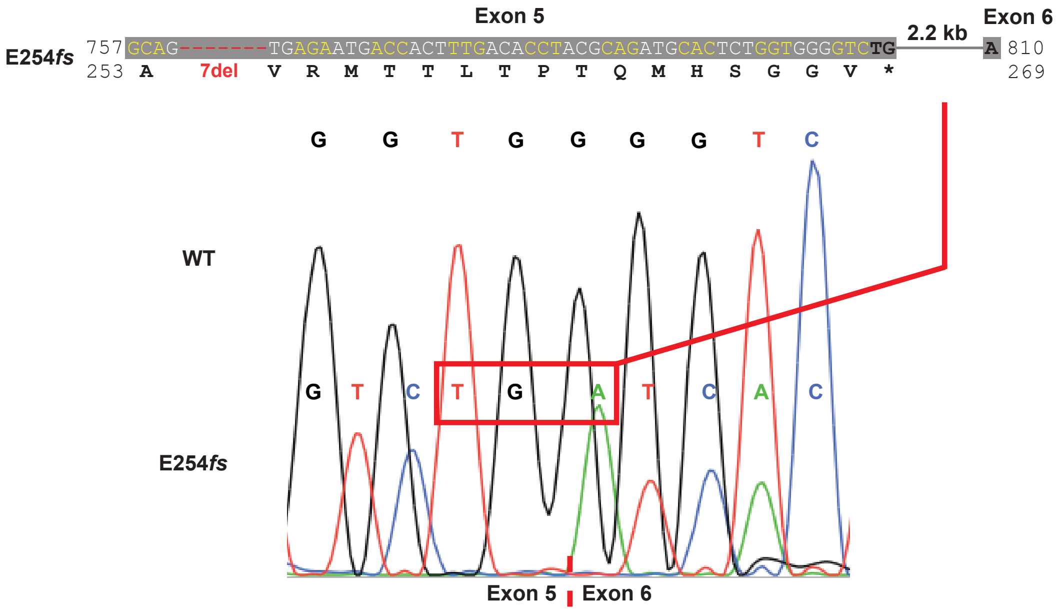

Figure 4—figure supplement 1

bioRxiv preprint doi: https://doi.org/10.1101/2024.01.04.574177; this version posted January 11, 2024. The copyright holder for this preprint (which was not certified by peer review) is the author/funder, who has granted bioRxiv a license to display the preprint in perpetuity. It is made available under aCC-BY 4.0 International license.

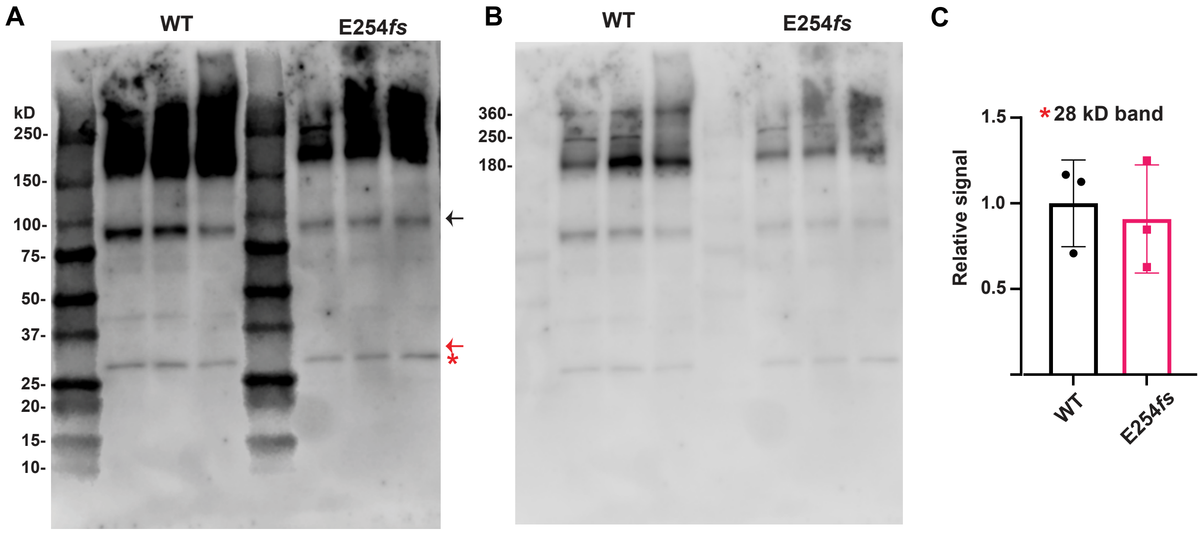

Figure 4—figure supplement 2

bioRxiv preprint doi: https://doi.org/10.1101/2024.01.04.574177; this version posted January 11, 2024. The copyright holder for this preprint (which was not certified by peer review) is the author/funder, who has granted bioRxiv a license to display the preprint in perpetuity. It is made available under aCC-BY 4.0 International license.

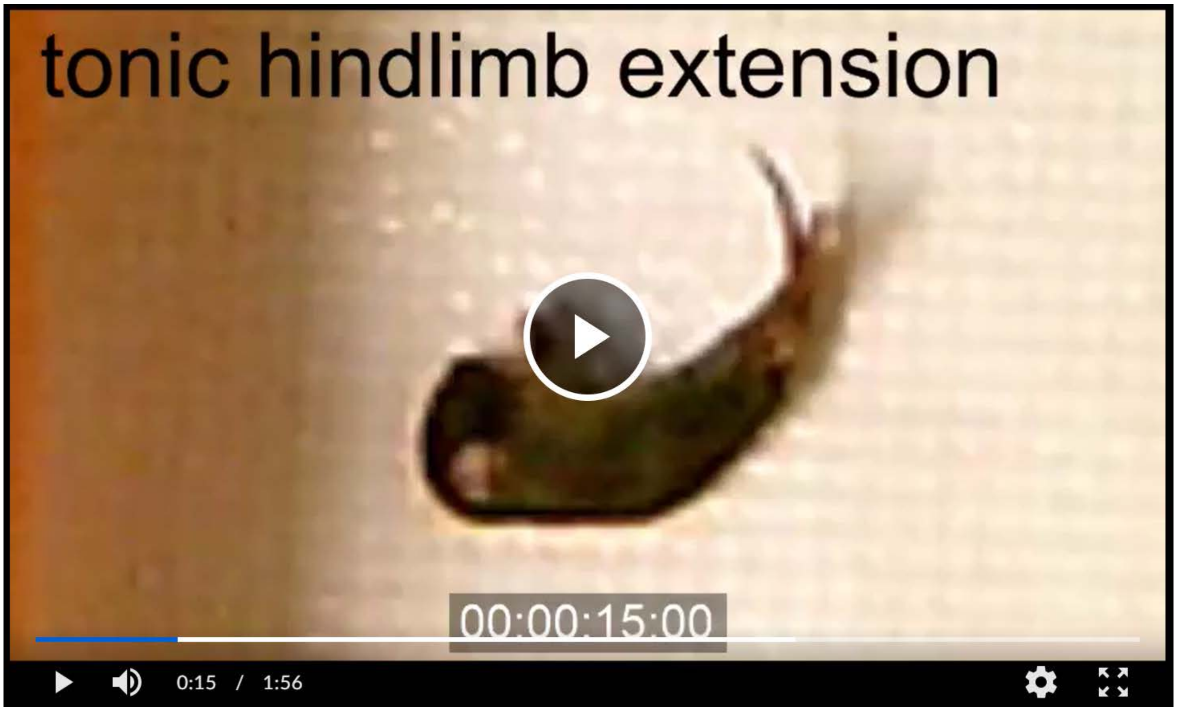

https://bcm.box.com/s/ig5nwnf86jor53vmpgofd22ehej2xae1

Figure 4—figure supplement 3

bioRxiv preprint doi: https://doi.org/10.1101/2024.01.04.574177; this version posted January 11, 2024. The copyright holder for this preprint (which was not certified by peer review) is the author/funder, who has granted bioRxiv a license to display the preprint in perpetuity. It is made available under aCC-BY 4.0 International license.

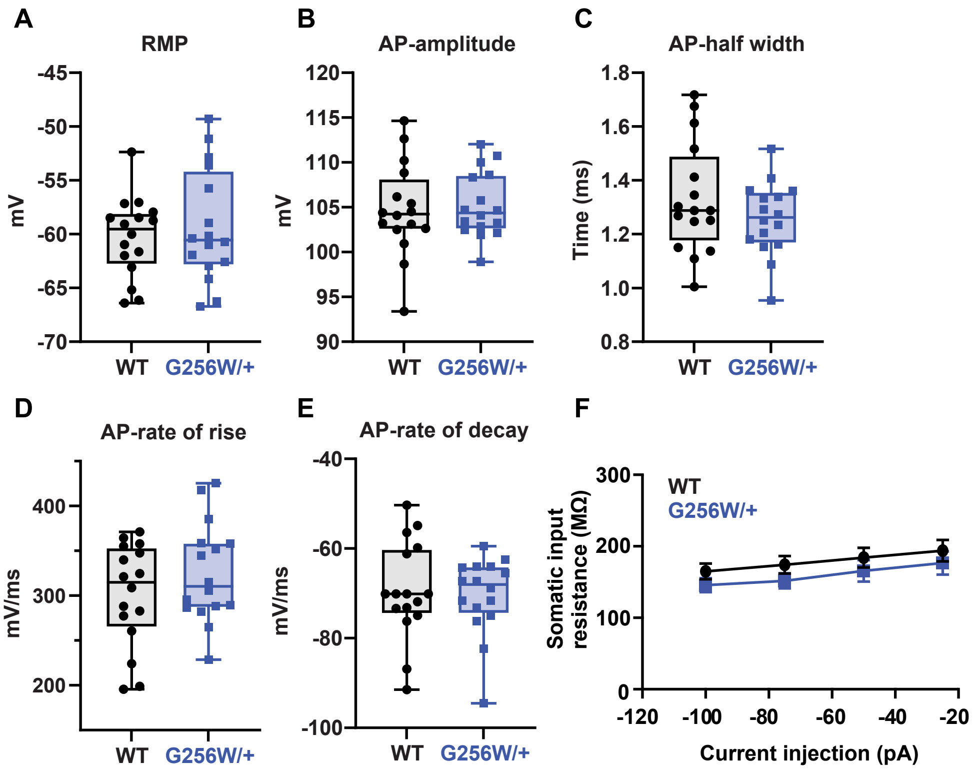

Figure 5—figure supplement 1

bioRxiv preprint doi: https://doi.org/10.1101/2024.01.04.574177; this version posted January 11, 2024. The copyright holder for this preprint (which was not certified by peer review) is the author/funder, who has granted bioRxiv a license to display the preprint in perpetuity. It is made available under aCC-BY 4.0 International license.

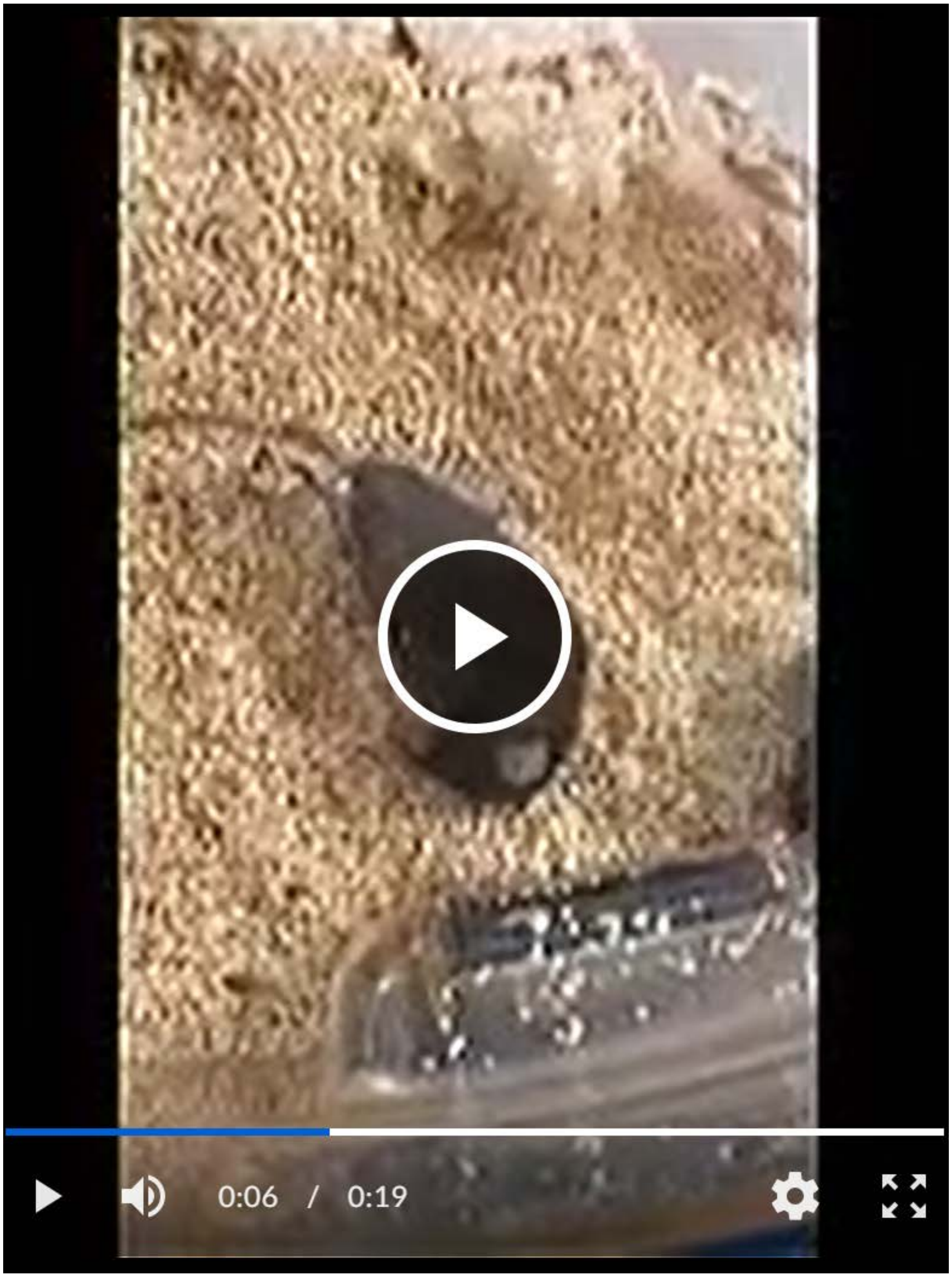

https://bcm.box.com/s/b8zd0xarcj332wupu7r618mv8l6y77jo

Figure 6—figure supplement 1

bioRxiv preprint doi: https://doi.org/10.1101/2024.01.04.574177; this version posted January 11, 2024. The copyright holder for this preprint (which was not certified by peer review) is the author/funder, who has granted bioRxiv a license to display the preprint in perpetuity. It is made available under aCC-BY 4.0 International license.

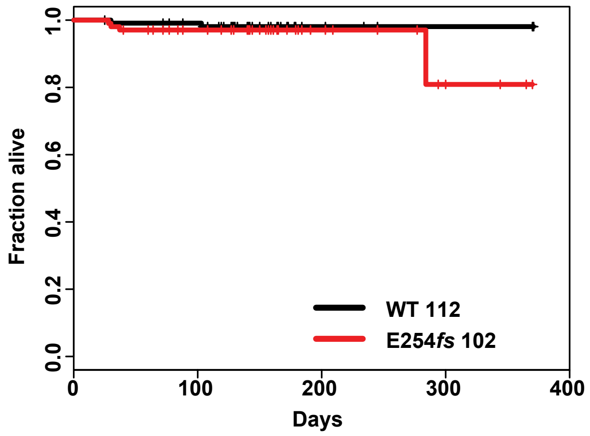

Figure 6—figure supplement 2

bioRxiv preprint doi: https://doi.org/10.1101/2024.01.04.574177; this version posted January 11, 2024. The copyright holder for this preprint (which was not certified by peer review) is the author/funder, who has granted bioRxiv a license to display the preprint in perpetuity. It is made available under aCC-BY 4.0 International license.

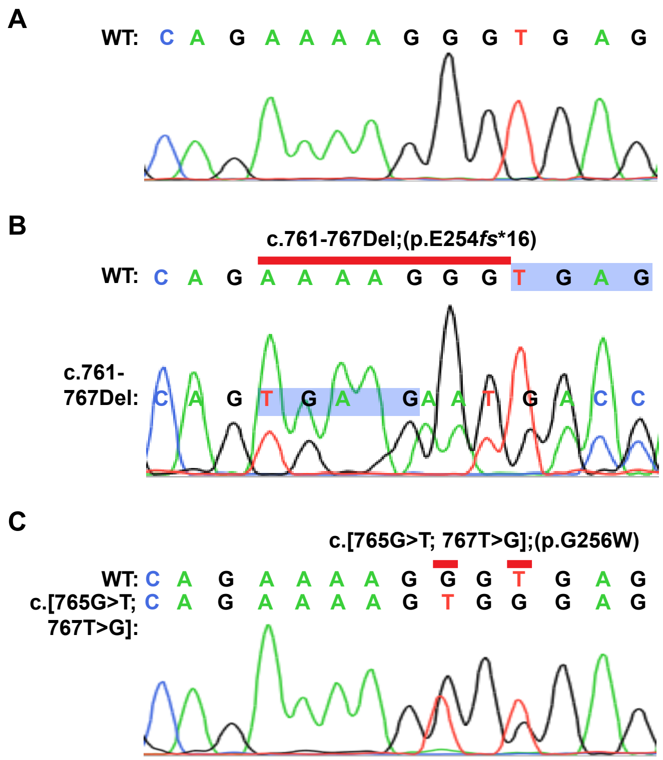

Figure 7—figure supplement 1

bioRxiv preprint doi: https://doi.org/10.1101/2024.01.04.574177; this version posted January 11, 2024. The copyright holder for this preprint (which was not certified by peer review) is the author/funder, who has granted bioRxiv a license to display the preprint in perpetuity. It is made available under aCC-BY 4.0 International license.

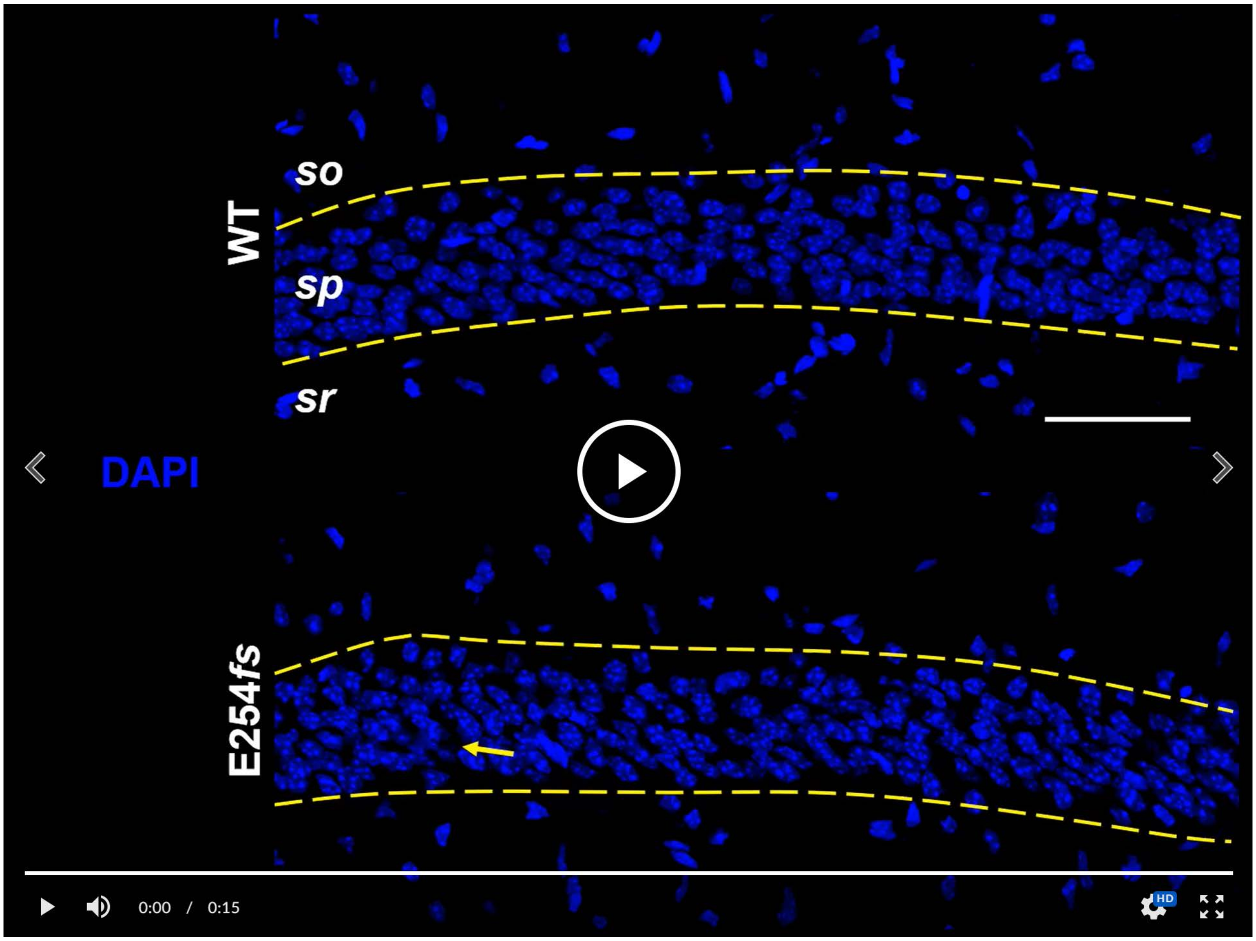

https://bcm.box.com/s/x608born0nxuaup3iu1ihyx239wacxv3

Figure 8—figure supplement 1

bioRxiv preprint doi: https://doi.org/10.1101/2024.01.04.574177; this version posted January 11, 2024. The copyright holder for this preprint (which was not certified by peer review) is the author/funder, who has granted bioRxiv a license to display the preprint in perpetuity. It is made available under aCC-BY 4.0 International license.

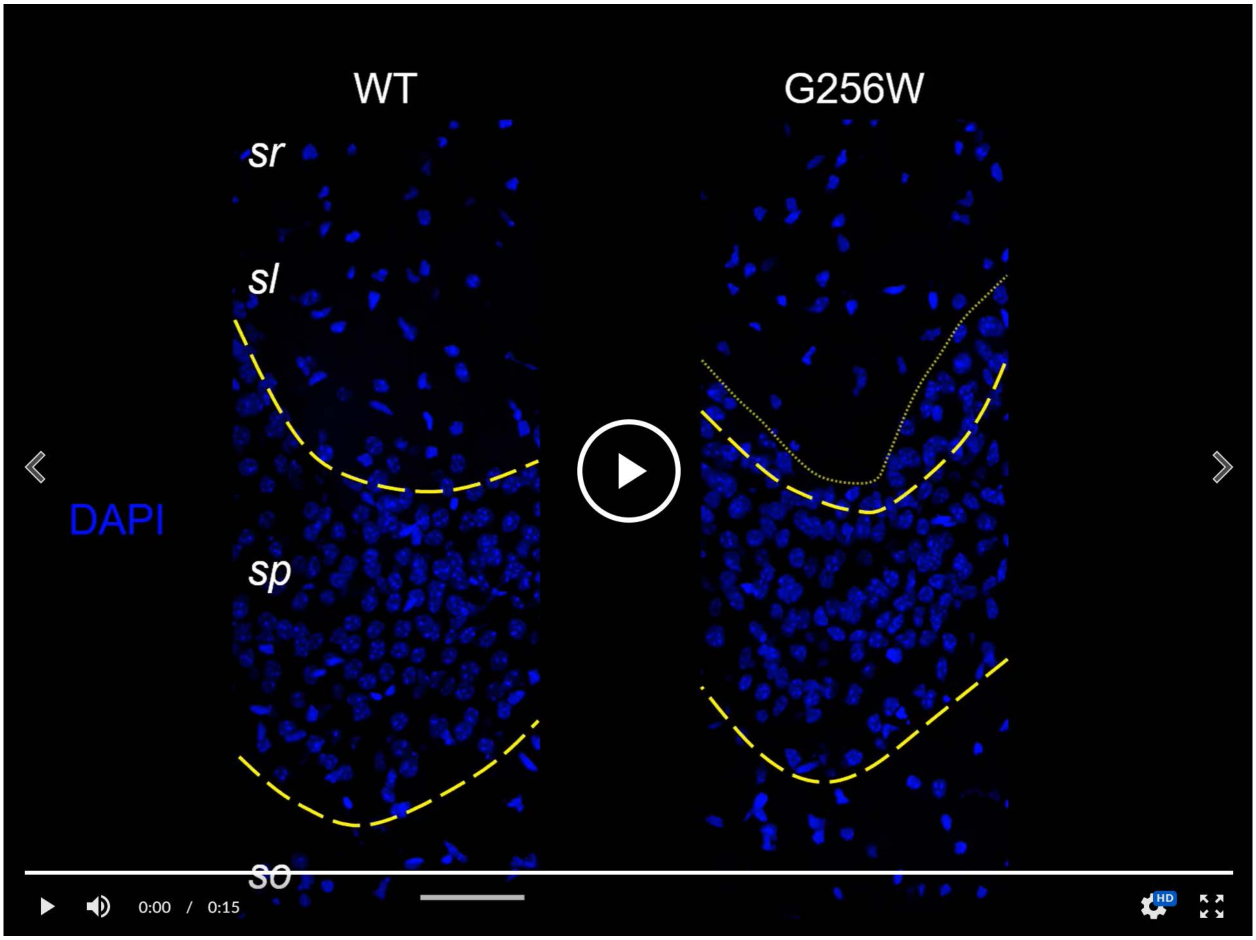

https://bcm.box.com/s/o4r8dpjloatvbkavhs4o3aklc8nvndta

Figure 8—figure supplement 2

bioRxiv preprint doi: https://doi.org/10.1101/2024.01.04.574177; this version posted January 11, 2024. The copyright holder for this preprint (which was not certified by peer review) is the author/funder, who has granted bioRxiv a license to display the preprint in perpetuity. It is made available under aCC-BY 4.0 International license.

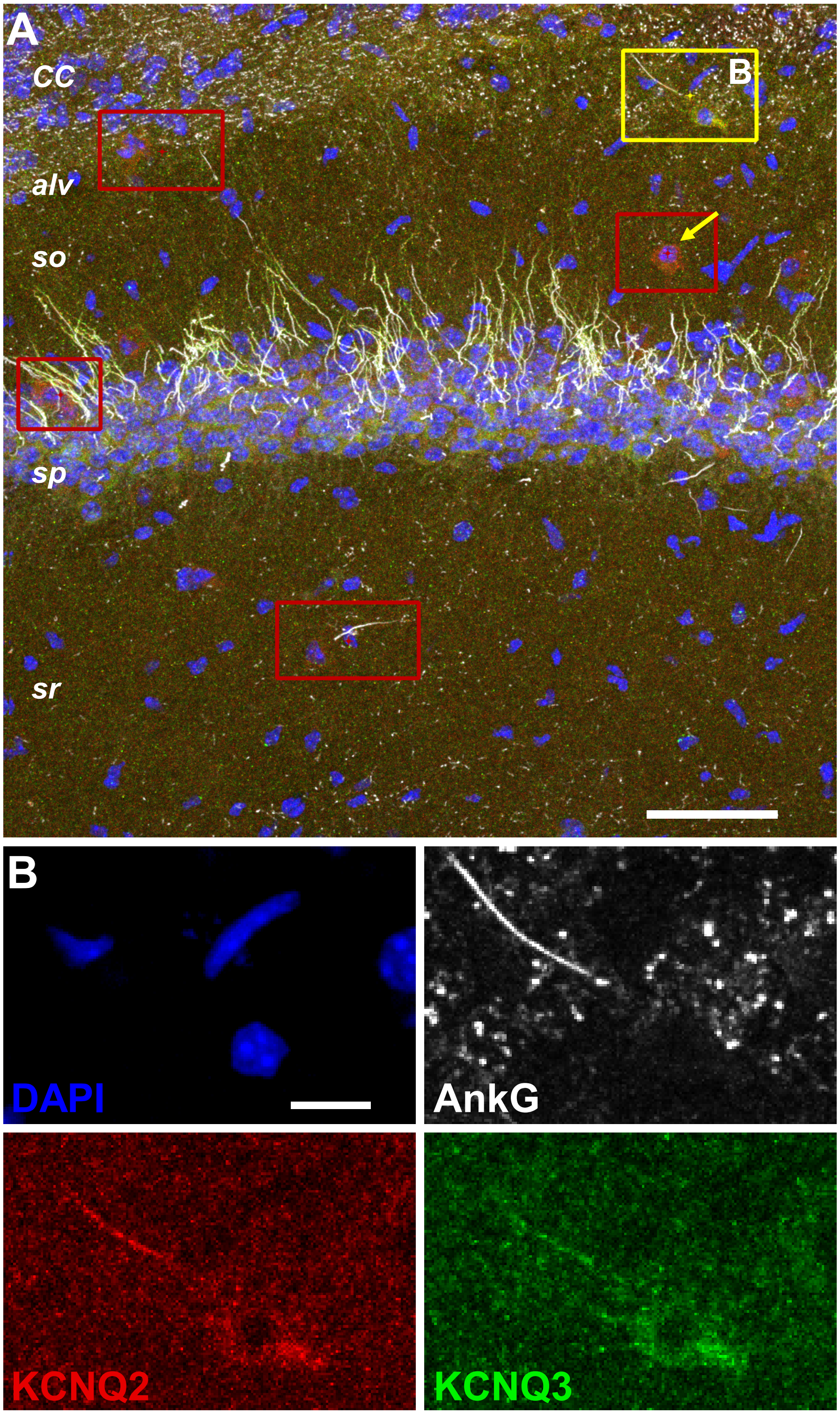

Figure 8—figure supplement 3

bioRxiv preprint doi: https://doi.org/10.1101/2024.01.04.574177; this version posted January 11, 2024. The copyright holder for this preprint (which was not certified by peer review) is the author/funder, who has granted bioRxiv a license to display the preprint in perpetuity. It is made available under aCC-BY 4.0 International license.

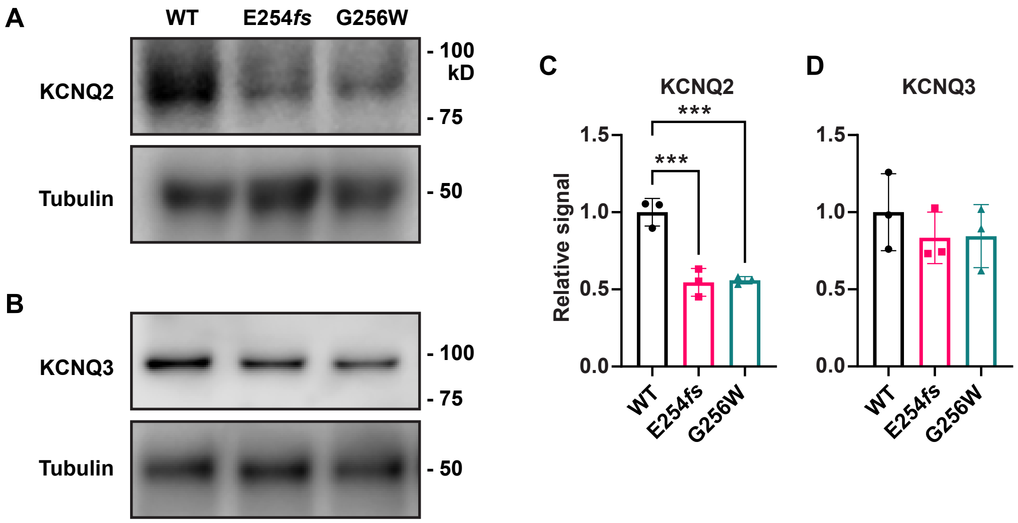

Figure 9—figure supplement 1

bioRxiv preprint doi: https://doi.org/10.1101/2024.01.04.574177; this version posted January 11, 2024. The copyright holder for this preprint (which was not certified by peer review) is the author/funder, who has granted bioRxiv a license to display the preprint in perpetuity. It is made available under aCC-BY 4.0 International license.

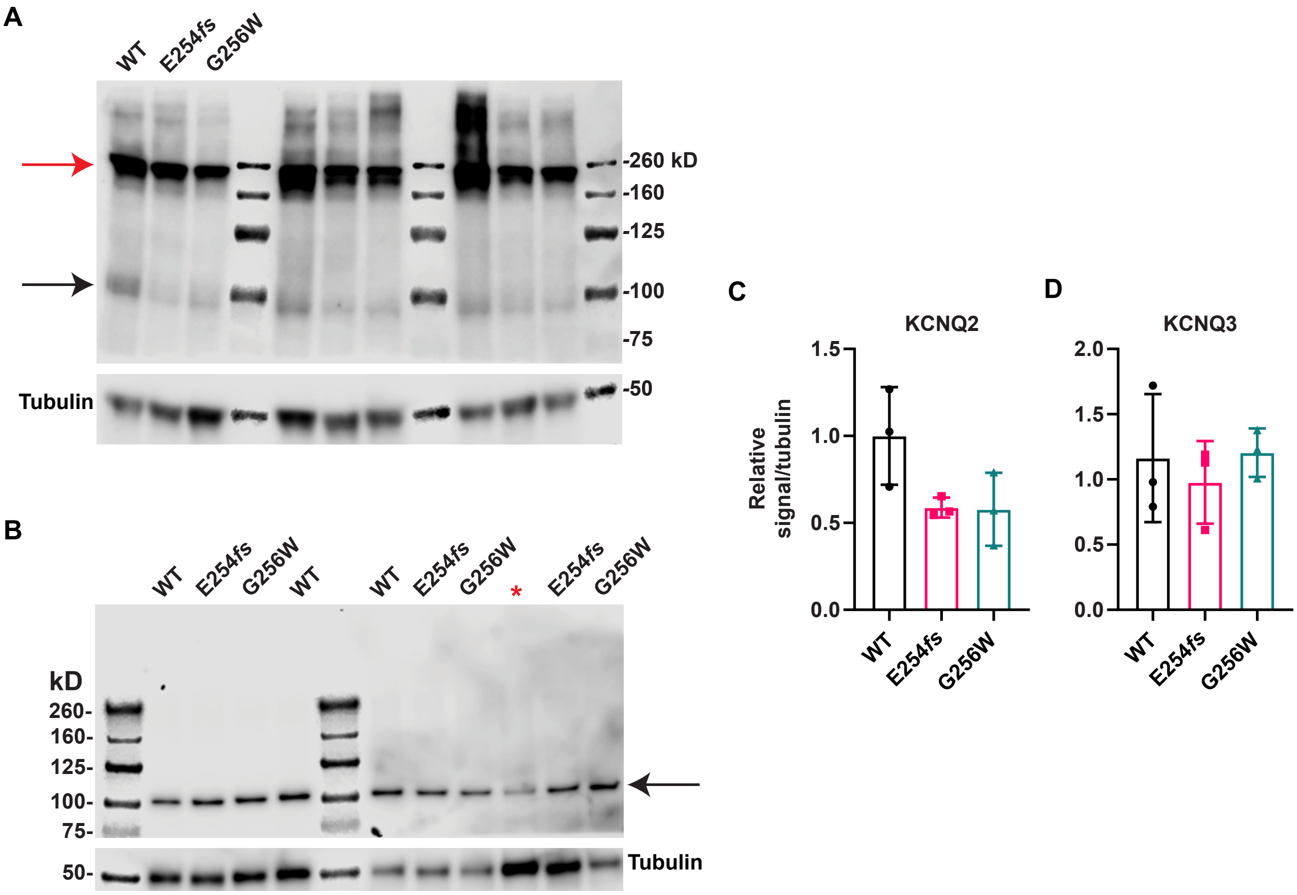

Figure 9—figure supplement 2
